# Time Modulation-Based Multi-User Covert Communication

**DOI:** 10.3390/e28070773

**Published:** 2026-07-08

**Authors:** Lanxiang Jiang, Xuanya Zhang, Qun Chen, Xin Wan, Fei Yang, Gang Yang

**Affiliations:** 1 School of Information and Communication Engineering, Communication University of China, Beijing 100024, China; lovejianglx@cuc.edu.cn (L.J.); zxy_0102@cuc.edu.cn (X.Z.); qunchen@cuc.edu.cn (Q.C.); yangsher@cuc.edu.cn (F.Y.); 2 State Key Laboratory of Media Convergence and Communication, Communication University of China, Beijing 100024, China; wallin82@cuc.edu.cn

**Keywords:** covert communication, time-modulated array (TMA), space-division multiple access (SDMA), harmonic beamforming

## Abstract

Multi-antenna-based covert communication techniques exploit spatial degrees of freedom to improve transmission efficiency under covertness constraints, but this generally comes at the cost of increased hardware complexity and power consumption. To this end, time-modulated arrays (TMA) enable multi-user covert communication with a single radio-frequency (RF) chain, providing a promising solution for low-complexity and energy-efficient covert communication. However, the infinite-order harmonics generated by time modulation spread signal energy over the entire spectrum, allowing the warden to enhance detection capability via cross-band observations, which aggravates signal leakage toward unintended directions. This paper develops a binary hypothesis testing model from the perspective of the warden based on infinite-order harmonic characteristics, to characterize the statistical properties and power distribution of harmonic-induced leakage. Furthermore, since the Kullback–Leibler (KL) divergence is intractable under infinite-order harmonic conditions, a computable upper bound is derived to enable covert constraint analysis. Considering the strong coupling among system parameters, an optimization problem is formulated to maximize the minimum covert transmission rate, and a genetic algorithm (GA) is employed for the joint design of time modulation, power allocation, and spatial phase. Simulation results demonstrate that the proposed scheme effectively suppresses signal leakage and improves covert transmission performance.

## 1. Introduction

Massive numbers of devices within the Internet of Things (IoT) exchange sensing, monitoring, and control information over open wireless channels. Such data typically contains highly sensitive information, such as user health status, location data, and critical system operating parameters, rendering communication security an increasingly critical and challenging concern [1,2,3]. Commonly adopted security mechanisms can be broadly classified into upper-layer encryption techniques [4] and physical layer security (PLS) [5,6,7]. Specifically, upper-layer encryption ensures confidentiality through sophisticated cryptographic protocols and algorithmic designs [8], whereas PLS exploits the inherent randomness and uniqueness of wireless channels, combined with signal processing and coding techniques, to achieve secure transmission [9]. However, both approaches fundamentally rely on the assumption that the transmitted signal has already been detected, and mitigate warden risks by concealing or protecting the information content. In many security-sensitive IoT applications, merely hiding the information content remains insufficient, since the existence of a communication activity itself may reveal critical system states or operational intentions. In industrial IoT, for instance, wireless links are commonly employed to transmit sensing, monitoring, and control information of industrial equipment [10]. Once these transmissions are successfully eavesdropped on or identified through traffic detection, attackers may infer critical production processes, equipment operating states, or control commands, thereby threatening control stability and operational safety [11]. Similarly, in smart grid systems, critical functions such as load frequency control rely on open communication networks to transmit measurement data and control commands, while the associated measurement and control channels may be vulnerable to cyberattacks such as false data injection, thereby compromising stable system operation [12]. Therefore, in such scenarios, even if attackers are unable to decrypt the specific information content, they may still infer system operating states, control actions, or changes in critical services by detecting the existence of communication activities, thereby launching further targeted attacks. Moreover, in communication scenarios with a high probability of line-of-sight links, signals are more susceptible to detection and interception by potential adversaries, thereby exposing the existence of communication [13]. Consequently, conventional security mechanisms alone are inadequate to meet the stringent security requirements of future wireless systems [14].

Covert communication aims to hide transmission activities from detection, thereby precluding further signal analysis by potential wardens, which fundamentally differs from conventional security methods that focus on protecting information content [15,16]. Early studies [17] established the square-root law for covert transmission over additive white Gaussian noise (AWGN) channels, indicating that, under covert constraints, the amount of reliably transmitted information scales only with the square root of the number of channel uses. This fundamental result implies that the achievable transmission rate is inherently limited under stringent covert requirements. Consequently, enhancing the reliability and transmission efficiency of the legitimate link while satisfying detection constraints has become a central problem in covert communication [18,19]. In this context, multi-antenna techniques have been shown to be effective by providing additional spatial degrees of freedom, thereby improving spatial controllability and reducing the detection capability of the warden [20,21,22]. Furthermore, as antenna arrays evolve from fixed-position architectures to movable ones, antenna positions have been incorporated into covert communication designs as additional optimization variables, thereby providing new position-domain degrees of freedom [23]. For multiuser covert communication, transmit beamforming and two-dimensional antenna positions can be jointly optimized under noise uncertainty to maximize the covert sum rate [24]. MA-enabled covert dual-functional radar-communication has also been investigated by jointly designing transmit beamforming, receive filtering, and antenna placement under radar SINR and covertness constraints [25]. However, due to the requirement of multiple radio-frequency (RF) chains, the practical deployment of multi-antenna techniques in covert communication remains constrained by increased hardware complexity and power consumption [26].

Time-modulated arrays (TMA) leverage an additional temporal degree of freedom beyond the spatial domain, enabling flexible beamforming with reduced hardware complexity and power consumption [27,28,29], thereby making TMA a promising solution for covert communication. In [30], time modulation was first incorporated into retrodirective arrays, leading to the time-modulated retrodirective array and a joint optimization framework for array patterns, transmit power, and packet length. Subsequently, the covert age of information (CAoI) metric was proposed in [31] to jointly characterize covertness and timeliness, and was minimized via power and packet length optimization. It has been shown that TMA achieves superior CAoI performance compared to phased arrays under similar covert constraints while requiring lower hardware complexity and power consumption [32]. These advantages have further enabled TMA-based covert communication in aerial platforms and UAV networks, where directional gain control can be achieved with low complexity [33]. Moreover, movable TMA extends the design space by introducing antenna positions as optimization variables, enabling joint optimization of transmission parameters to maximize covert throughput [26]. However, time modulation inherently generates infinite-order harmonics, causing signal energy to spread over the entire spectrum [34,35]. Under cross-band detection or non-carrier frequency monitoring, covert designs based on finite-order harmonic models fail to suppress leakage effectively, resulting in inevitable signal exposure in unintended directions. While existing studies primarily rely on finite-harmonic assumptions, the performance of TMA-based covert communication under practical infinite-order harmonic conditions remains largely unexplored. Since covert communication fundamentally requires minimizing detectability, such harmonic-induced leakage may significantly degrade system covertness. Therefore, performance analysis and optimization of TMA-based covert communication under infinite-order harmonic conditions remain urgently needed.

In this context, this paper investigates a time-modulated array-based space-division multiple access (TMA-SDMA)covert communication system under infinite-order harmonic signals and develops a performance analysis and optimization framework that explicitly accounts for harmonic-induced leakage. In contrast to conventional approaches based on fundamental signals assumptions, the proposed method captures the spectral spreading effect caused by time modulation, enabling a more accurate characterization of covert performance limits and transmission efficiency, and providing theoretical guidance for the design of TMA-SDMA covert systems. The main contributions are summarized as follows.

- TMA-Based Multi-User Covert Communication: We propose a TMA-based multi-user covert communication scheme under a single-RF-chain architecture, where time modulation is exploited to support SDMA transmission while reducing hardware complexity. Different from conventional approaches based on fundamental-component or finite-harmonic assumptions, the proposed analysis models Willie’s received signal using infinite-order harmonic components, and thus reveals the statistical characteristics and power distribution of high-order harmonic-induced leakage.
- Closed-Form KL-Divergence Bound Under Infinite Harmonics: The exact Kullback–Leibler (KL) divergence under infinite-order harmonics is analytically intractable due to the accumulated contributions of all harmonic components. To address the challenge, an equivalent closed-form upper bound on the KL divergence is derived. The bound provides a tractable covert constraint for performance evaluation and optimization, enabling more comprehensive assessment of covert performance than models relying only on finite or fundamental harmonic components.
- KL-Divergence Bound-Based Joint Optimization Design: Based on the derived KL-divergence upper bound, a max-min optimization problem is formulated to maximize the minimum covert transmission rate among multiple users under the covertness constraint. A genetic algorithm (GA)-based joint design is developed to optimize the time-modulation sequence, power allocation, and spatial phase parameters. The optimization framework directly accounts for infinite-harmonic leakage, thereby offering design guidance for practical TMA-SDMA covert communication systems.
- Numerical Validation: Numerical simulations validate the effectiveness of the derived KL-divergence upper bound and the proposed joint optimization scheme. Comparisons between the fundamental-component model and the infinite-order harmonic model reveal the performance deviation caused by neglecting high-order harmonics. The results show that the proposed design improves covert transmission performance while suppressing signal leakage toward the warden, confirming the importance of infinite-harmonic-aware modeling for accurate covert performance assessment.

The structure of the subsequent sections is organized as follows. Section 2 introduces the system model, including the transmit signal model at Alice, the received signal model at legitimate users, and the binary hypothesis testing model at Willie. Section 3 presents the covert performance analysis, focusing on the derivation of covert constraints and the upper bound of the KL divergence under cross-band detection. Section 4 formulates the max–min transmission rate optimization problem and develops the proposed genetic algorithm-based joint design. Section 5 provides numerical results to validate the proposed scheme and analyzes the impact of covert constraints and system parameters. Finally, Section 6 concludes with a summary of the key findings in this paper.

Notations: Notations used in this paper are summarized as follows. Scalars are denoted by italic lowercase letters, vectors by bold lowercase letters, and matrices by bold uppercase letters. For a complex scalar *a*, $\left|a\right|$ and $a^{*}$ denote its absolute value and conjugation, respectively. For a complex vector a, $∥a∥$ denotes its Euclidean norm. For a matrix A, ${\left[A\right]}_{ij}$, $A^{-1}$, $A^{T}$, and $A^{H}$ denote the $\left(i,j\right)$-th entry, inverse, transpose, and conjugate transpose, respectively; $\det\left(A\right)$ denotes its determinant.

## 2. System Model

**Figure 1:**
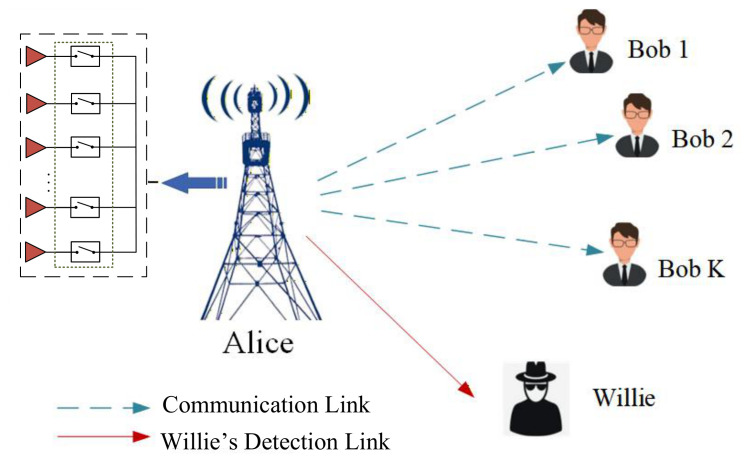
System model of TMA-based multi-user SDMA covert communication.

This section presents the system model of a TMA-based SDMA downlink covert communication, comprising a base station (Alice), *K* single-antenna legitimate users (Bobs), and a warden (Willie), as illustrated in Figure 1. The base station employs a uniform linear TMA with *N* elements to serve Bobs via SDMA. Meanwhile, a single-antenna Willie attempts to detect the presence of transmission, posing a threat to system covertness. Notably, TMA produces multi-order harmonic beams and sidelobes in non-user directions, leading to unintended radiation that can be exploited by Willie for detection.

### 2.1. Transmit Signal Model at Alice

**Figure 2:**
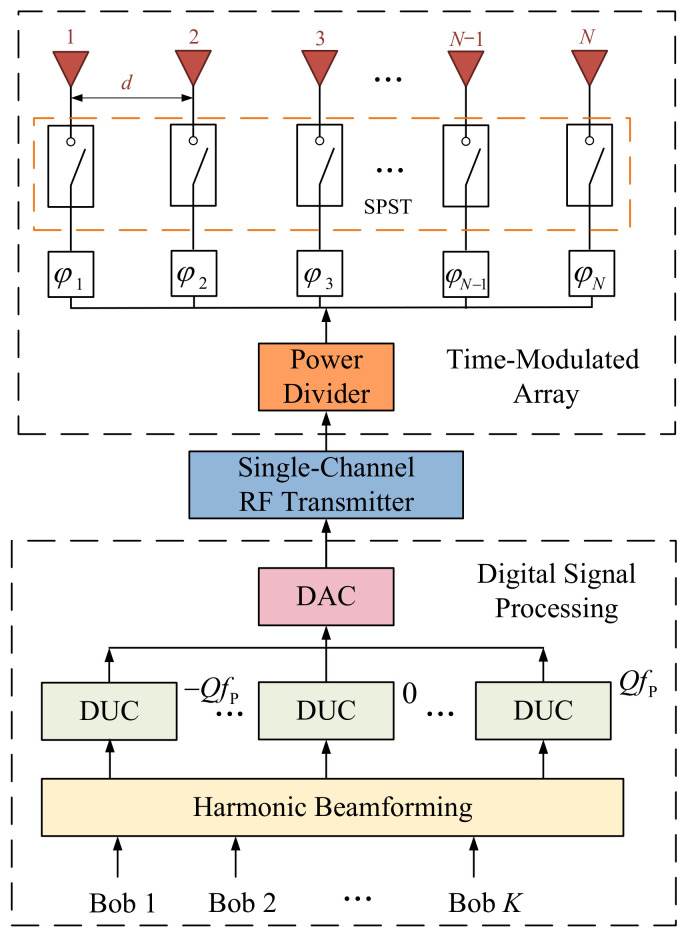
Block diagram of the harmonic-based SDMA transceiver in the downlink scenario.

As shown in Figure 2, Alice employs a TMA-based array architecture [36]. The array operates at the carrier frequency $f_{C}$ with a broadside direction perpendicular to the ground. The element spacing is d=λ/2, where λ denotes the carrier wavelength. Consider *K* legitimate Bobs, indexed by $k\in K≜\left\{1,2,\cdots ,K\right\}$, corresponding to *K* independent data streams. Alice transmits with total power $P_{s}$, which is allocated across users via $\rho_{k}$, satisfying $\sum_{k}\rho_{k}=1$. The transmit signal vector is defined as $s={\left[s_{1},s_{2},\cdots ,s_{K}\right]}^{T}$ with $E\left(ss^{H}\right)=I_{K}$, yielding a per-user transmit power of $\rho_{k}P_{s}$. Alice exploits harmonics from the −Q-th to the +Q-th order, resulting in 2Q+1 harmonics carrying useful information. Let $w_{k,q}$ denote the precoding coefficient associated with the *q*-th harmonic for user *k*. The corresponding beamforming vector for Bob *k* can be expressed as

(1) $$w_{k}={\left[w_{k,-Q},\cdots ,w_{k,Q}\right]}^{T}.$$

The precoded baseband signals for all Bobs are transferred to relevant harmonic frequencies utilizing digital up-converters (DUCs) and combined to a single-channel complex baseband signal. And then the signals are passed through a digital-to-analog converter (DAC), followed by modulation via a single RF chain. The resulting RF signal at the *q*-th harmonic is given by

(2) $$x_{q}\left(t\right)=\sum_{k=1}^{K}\sqrt{\rho_{k}P_{s}}w_{k,q}s_{k}e^{j2\pi \left(f_{C}+qf_{P}\right)t},$$

where $f_{P}$ is the time modulation frequency. The RF signal is then fed into a power divider and equally distributed to the RF front-ends of each antenna element. Each path first undergoes phase delay processing via a phase shifter, followed by periodic time modulation executed by an independently controlled single-pole-single-throw (SPST) switch. Accordingly, the transmitted signal at the *n*-th antenna element can be expressed as

(3) $$x_{n}^{\mathrm{RF}}\left(t\right)=\frac{1}{\sqrt{N}}e^{j\varphi_{n}}U_{n}\left(t\right)\sum_{q=-Q}^{Q}x_{q}\left(t\right),$$

where $U_{n}\left(t\right)$ denotes the time modulation function applied at the *n*-th element, defined as

(4) $$U_{n}\left(t\right)=\left\{\begin{array}{cc} 1, & t_{\mathrm{on},n}+pT_{P}\leq t<t_{\mathrm{off},n}+pT_{P},\\ 0, & \mathrm{otherwise}. \end{array}\right.$$

Within each modulation period $T_{P}$ (for integer p∈Z), $U_{n}\left(t\right)$ represents a periodic switching process, where the switch is turned on at $t_{\mathrm{on},n}+pT_{P}$ and turned off at $t_{\mathrm{off},n}+pT_{P}$, remaining off otherwise. Owing to periodicity, $U_{n}\left(t\right)$ admits a Fourier series expansion as

(5) $$U_{n}\left(t\right)=\sum_{q^{\prime }=-\infty }^{\infty }\alpha_{n,q^{\prime }}e^{j2\pi q^{\prime }f_{P}t}$$

where $q^{\prime }\in Z$ is the harmonic index. The associated Fourier coefficient at the $q^{\prime }$-th harmonic for the *n*-th antenna element is denoted by $\alpha_{n,q^{\prime }}$, whose expression is characterized by

(6) $$\begin{array}{cc} \alpha_{n,q^{\prime }}\; & =\frac{1}{T_{P}}\int_{0}^{T_{P}}U_{n}\left(t\right)e^{-j2\pi q^{\prime }f_{p}t}\;dt\\ \; & =\tau_{n}\;\mathrm{sinc}\;\left(\pi q^{\prime }\tau_{n}\right)e^{-j2\pi q^{\prime }{\bar{\tau }}_{n}}. \end{array}$$

Here, τn=tn,off−tn,ontn,off−tn,onTPTP and τ¯n=tn,off+tn,ontn,off+tn,on2TP2TP represent the normalized duty cycle and switching midpoint, respectively. By substituting (5) into (3), the transmitted signal at the *n*-th antenna element is obtained as

(7) $$x_{n}\left(t\right)=\frac{1}{\sqrt{N}}e^{j\varphi_{n}}\sum_{q=-Q}^{Q}\left(\sum_{q^{\prime }=-\infty }^{\infty }\alpha_{n,q^{\prime }}e^{j2\pi q^{\prime }f_{P}t}\right)x_{q}\left(t\right).$$

From the above, the signal at the $\left(q^{\prime }=-q\right)$-th time-modulation harmonic is mapped to the center frequency $f_{C}$. The equivalent signal transmitted by the *n*-th element at $f_{C}$ is given by

(8) $$x_{n,f_{C}}\left(t\right)=\frac{1}{\sqrt{N}}e^{j\varphi_{n}}\sum_{q=-Q}^{Q}\alpha_{n,-q}\sum_{k=1}^{K}\sqrt{\rho_{k}P_{s}}w_{k,q}s_{k}e^{j2\pi f_{C}t}.$$

### 2.2. Received Signal Model at Bobs

Due to the time modulation, the transmitted signal contains multiple harmonic components, which jointly contribute to the received signals at Bobs. The channel from Alice to the *k*-th Bob is given by

(9) $$h_{\mathrm{AB},k}=h_{\mathrm{AB},k}{\left[1,e^{-j2\pi \frac{d}{\lambda }\sin\left(\theta_{k}\right)},\cdots ,e^{-j2\pi \left(N-1\right)\frac{d}{\lambda }\sin\left(\theta_{k}\right)}\right]}^{T}$$

where $\theta_{k}$ denotes the angle of departure (AoD) from Alice to the *k*-th Bob, and $h_{\mathrm{AB},k}$ denotes the channel gain between Alice and the *k*-th Bob. Accordingly, the wireless channel between Alice and Bobs can be expressed as $H_{\mathrm{AB}}={\left[h_{\mathrm{AB},1},h_{\mathrm{AB},2},\cdots ,h_{\mathrm{AB},K}\right]}^{T}$. Assuming that all Bobs operate at the carrier frequency $f_{C}$, and based on (8), the received signal at Bob *k* after filtering, downconversion, and baseband sampling is represented as

(10) $$y_{k}=\frac{1}{\sqrt{N}}\sum_{n=1}^{N}h_{\mathrm{AB},n,k}e^{j\varphi_{n}}\sum_{q=-Q}^{Q}\alpha_{n,-q}\sum_{k=1}^{K}\sqrt{\rho_{k}P_{s}}w_{k,q}s_{k}+n_{k}$$

where $n_{k}∼\mathrm{CN}\left(0,\sigma_{k}^{2}\right)$ denotes the AWGN at Bob *k*, and $h_{\mathrm{AB},n,k}$ denotes the channel from the *n*-th TMA element at Alice to Bob *k*. Let $P≜\mathrm{diag}\left(\sqrt{\rho_{1}},\sqrt{\rho_{2}},\cdots ,\sqrt{\rho_{K}}\right)$ denote the power allocation, and $\Phi ≜\mathrm{diag}\left(e^{j\varphi_{1}},e^{j\varphi_{2}},\cdots ,e^{j\varphi_{N}}\right)$ denote the phase-shift matrix. By employing vector notation to represent the received signals of all Bobs, the overall received signal model can be expressed as

(11) $$y=\sqrt{\frac{P_{s}}{N}}H_{\mathrm{AB}}\Phi \mathrm{BWPs}+n$$

where $W=\left[w_{1},w_{2},\cdots ,w_{K}\right]$ denote the precoding matrices, and $n={\left[n_{1},n_{2},\cdots ,n_{K}\right]}^{T}$ is independent of the data vector s. The matrix B is the harmonic characteristic matrix determined by the Fourier coefficients of the time modulation functions, given by

(12) $$B=\left[\begin{array}{cccc} \alpha_{1,Q} & \alpha_{1,Q-1} & \cdots & \alpha_{1,-Q}\\ \alpha_{2,Q} & \alpha_{2,Q-1} & \cdots & \alpha_{2,-Q}\\ \vdots & \vdots & \ddots & \vdots \\ \alpha_{N,Q} & \alpha_{N,Q-1} & \cdots & \alpha_{N,-Q} \end{array}\right].$$

From (11), $H_{\mathrm{AB}}\Phi B$ represents the equivalent channel in the harmonic domain. A zero-forcing (ZF) precoding scheme is adopted to suppress multi-user interference, with the precoder given by

(13) $$W={\left(H_{\mathrm{AB}}\Phi B\right)}^{H}{\left(H_{\mathrm{AB}}\Phi B{\left(H_{\mathrm{AB}}\Phi B\right)}^{H}\right)}^{-1}.$$

It should be noted that ZF precoding is adopted as a representative linear precoding scheme to suppress multi-user interference and maintain the analytical tractability of the proposed TMA-SDMA covert transmission model. The feasibility of ZF precoding requires the effective multi-user channel matrix to have sufficient rank, i.e., the available spatial/harmonic degrees of freedom should be no smaller than the number of served users. For analytical tractability, perfect channel state information (CSI)is assumed in this paper to characterize the performance upper bound of the harmonic-based beamforming design. In practical systems, $H_{\mathrm{AB}}$ can be obtained through pilot-assisted channel estimation or CSI feedback [37]. For TMA-based transceivers, time modulation maps the physical channels to equivalent harmonic-domain channels, while the time-modulation sequence, harmonic characteristic matrix B, and phase-shift matrix Φ are generated and known at Alice [28]. Therefore, once Alice–Bob CSI is available, the equivalent channel $H_{\mathrm{AB}}\Phi B$ can be constructed from the estimated physical CSI and the known TMA parameters.

### 2.3. Binary Detection Model at Willie

From (7), time modulation introduces infinite harmonic components distributed around the carrier frequency $f_{C}$ with spacing $f_{P}$. Since Alice has no prior knowledge of the frequency bands monitored by Willie, designing the covert transmission strategy based only on a subset of harmonic components may leave residual signal energy at other frequencies that can still be exploited by Willie. Specifically, Willie’s objective is to determine whether the received signal contains only background noise or also includes components generated by Alice’s transmission. In a TMA-based SDMA system, periodic time modulation spreads the transmitted signal energy across multiple harmonic frequencies, such that detectable leakage is not confined to the carrier-frequency component. Consequently, even if the fundamental harmonic is effectively suppressed, higher-order harmonics may still carry statistical signatures of the transmission activity, thereby degrading the overall covertness performance.

To ensure the analysis remains valid for any possible detection band, a conservative worst-case detection model is first considered. In this model, Willie is assumed to have ideal reception and processing capabilities and can exploit all harmonic components generated by time modulation. This assumption is not intended to imply that a practical Willie can physically monitor an unbounded frequency range. In practical receivers, the observable harmonic components are limited by hardware bandwidth, receiver sensitivity, noise floor, and signal processing capability. Therefore, the finite-band detection case is first represented by a finite maximum observable harmonic order, and the infinite-harmonic model is obtained as its limiting case.

Accordingly, the equivalent baseband signal $y_{W}\left(t\right)$ received at Willie after downconversion is formulated as

(14) $$y_{W}\left(t\right)=\left\{\begin{array}{cc} n_{W}\left(t\right), & H_{0},\\ \sqrt{\frac{P_{s}}{N}}\sum_{n=1}^{N}\sum_{q=-Q}^{Q}\sum_{q^{\prime }=-L}^{L}\sum_{k=1}^{K}h_{\mathrm{AW},n,q^{\prime },q}e^{j\varphi_{n}}\alpha_{n,q^{\prime }}w_{k,q}\sqrt{\rho_{k}}s_{k}e^{j2\pi \left(q^{\prime }+q\right)f_{P}t}+n_{W}\left(t\right), & H_{1} \end{array}\right.$$

where $n_{W}∼CN\left(0,\sigma_{W}^{2}\right)$ denotes the AWGN at Willie, and *L* is the maximum harmonic order introduced by time modulation. The coefficient $h_{\mathrm{AW},n,q^{\prime },q}$ denotes the channel from the *n*-th antenna element to Willie at the $\left(q+q^{\prime }\right)$-th harmonic. Based on (14), for notational convenience, let $m=q+q^{\prime }$, where $m\in \left\{-M,-M+1,\cdots ,M\right\}\left(M=L+Q\right)$. In the following finite-dimensional representation, *M* denotes the maximum harmonic order included in Willie’s observation. For a practical finite-band Willie, *M* can be set as the maximum observable harmonic order determined by the receiver bandwidth and hardware capability. Increasing *M* means that Willie can collect more harmonic leakage components. The infinite-harmonic joint detection model corresponds to the limiting case M→∞, which provides a conservative benchmark for covertness evaluation. Under finite-band detection, fewer harmonic leakage components are available to Willie, and the resulting detection capability is no stronger than that under the infinite-harmonic assumption. The sampled received signal at Willie at the *m*-th harmonic is then given by

(15) $$y_{W,m}=\left\{\begin{array}{cc} n_{W,m}, & H_{0},\\ \sqrt{\frac{P_{s}}{N}}\sum_{n=1}^{N}h_{\mathrm{AW},n,m}e^{j\varphi_{n}}\sum_{q=-Q}^{Q}\alpha_{n,m-q}\sum_{k=1}^{K}w_{k,q}\sqrt{\rho_{k}}s_{k}+n_{W,m}, & H_{1}. \end{array}\right.$$

By stacking the harmonic components from order −M to *M* into a vector, the received signal at Willie can be rewritten as

(16) $$y_{W}=\left\{\begin{array}{cc} n_{W}, & H_{0},\\ \sqrt{\frac{P_{s}}{N}}\sum_{n=1}^{N}H_{\mathrm{AW},n}A_{n}\mathrm{WPs}+n_{W}, & H_{1}. \end{array}\right.$$

where $y_{W}={\left[y_{W,-M},y_{W,-M+1},\cdots ,y_{W,M}\right]}^{T}$ denotes the received signal vector at Willie. The diagonal matrix $H_{\mathrm{AW},n}=e^{j\varphi_{n}}\mathrm{diag}\left(h_{\mathrm{AW},n,-M},h_{\mathrm{AW},n,-M+1},\cdots ,h_{\mathrm{AW},n,M}\right)$ is defined to characterize the channel from the *n*-th antenna element at Alice to Willie across all harmonic frequencies. The vector $n_{W}∼CN\left(0,\sigma_{W}^{2}I_{\left(2M+1\right)}\right)$ denotes the AWGN at Willie. The matrix $A_{n}\in C^{\left(2M+1\right)\times \left(2Q+1\right)}$ represents the harmonic mixing matrix of the precoded transmit signal at the *n*-th antenna element after time modulation, whose expression is given by

(17) $$A_{n}=\left[\begin{array}{cccc} \alpha_{n,-M+Q} & \alpha_{n,-M+Q-1} & \cdots & \alpha_{n,-M-Q}\\ \alpha_{n,-M+Q+1} & \alpha_{n,-M+Q} & \cdots & \alpha_{n,-M-Q+1}\\ \vdots & \vdots & \ddots & \vdots \\ \alpha_{n,M+Q} & \alpha_{n,M+Q-1} & \cdots & \alpha_{n,M-Q} \end{array}\right]$$

where the (a,b)-th entry can be written as ${\left[A_{n}\right]}_{ab}=\alpha_{n,-M+Q+a-b}$, representing the Fourier coefficient corresponding to the $\left(-M+a-1\right)$-th observed frequency component induced by the $\left(b-Q-1\right)$-th harmonic of the precoded transmit signal after time modulation. The matrix $\sum_{n=1}^{N}H_{\mathrm{AW},n}A_{n}$ thus acts as the equivalent Alice–Willie channel, denoted by ${\tilde{H}}_{\mathrm{AW}}$, yielding the received signal model can be simplified as

(18) $$y_{W}=\left\{\begin{array}{cc} n_{W}, & H_{0},\\ \sqrt{\frac{P_{s}}{N}}{\tilde{H}}_{\mathrm{AW}}\mathrm{WPs}+n_{W}, & H_{1}. \end{array}\right.$$

The equivalent channel ${\tilde{H}}_{\mathrm{AW}}$ captures the combined effect of the Alice–Willie propagation channels and the harmonic mixing induced by time modulation. Therefore, it represents the aggregate spatial–harmonic leakage observed by Willie. Under this model, Willie’s task reduces to a binary hypothesis test, where $H_{0}$ denotes the absence of transmission from Alice to Bob, and $H_{1}$ denotes the presence of transmission. A larger leakage component associated with ${\tilde{H}}_{\mathrm{AW}}$ makes the received signals under $H_{0}$ and $H_{1}$ more statistically distinguishable, thereby improving Willie’s detection capability.

## 3. Covert Performance Analysis of TMA-SDMA

This section characterizes the received signal statistics at Willie and derives an upper bound on the KL divergence, which quantifies the covertness constraint under infinitely many harmonic components and provides the basis for subsequent optimization.

### 3.1. Covertness Constraint Analysis Based on KL Divergence

According to [14], the probability that Willie decides in favor of $H_{1}$ when $H_{0}$ is true is defined as the false alarm probability $P_{\mathrm{FA}}$, while the probability that Willie decides in favor of $H_{0}$ when $H_{1}$ is true is referred to as the miss detection probability $P_{\mathrm{MD}}$. Accordingly, the false alarm and miss detection probabilities are denoted by $P_{\mathrm{FA}}=\mathrm{Pr}\left\{D_{1}|H_{0}\right\}$ and $P_{\mathrm{MD}}=\mathrm{Pr}\left\{D_{0}|H_{1}\right\}$, respectively, where $D_{1}$ indicates that Willie declares the presence of transmission from Alice, and $D_{0}$ indicates the absence of transmission. The two probabilities $P_{\mathrm{FA}}$ and $P_{\mathrm{MD}}$ directly determine Willie’s detection error probability. For a given detector, the DEP is defined as $\xi =P_{\mathrm{FA}}+P_{\mathrm{MD}}$, where a larger ξ means that Willie has more difficulty distinguishing whether Alice is transmitting. Therefore, the covertness requirement is essentially imposed on Willie’s binary detection performance through these two probabilities. To ensure covertness, Willie is assumed to employ the optimal detector to determine whether Alice is transmitting to Bob. The corresponding decision rule at Willie is given by

(19) $$\frac{P_{1}≜f\left(y_{W}|H_{1}\right)}{P_{0}≜f\left(y_{W}|H_{0}\right)}\overset{D_{1}}{\underset{D_{0}}{≷}}1$$

where $P_{0}$ and $P_{1}$ denote the likelihood functions under hypotheses $H_{0}$ and $H_{1}$, respectively. Assuming the transmit signal follows a Gaussian distribution, and based on (18), when Alice is inactive, the received signal at Willie consists solely of AWGN, whereas when Alice is active, it consists of both the transmitted signal and noise. Accordingly, under both hypotheses, the received signal $y_{W}$ at Willie can be modeled as zero-mean complex Gaussian random vectors, given by

(20) $$y_{W}∼\left\{\begin{array}{cc} CN\left(0,\sigma_{W}^{2}I_{2M+1}\right), & H_{0},\\ CN\left(0,\frac{P_{s}}{N}{\tilde{H}}_{\mathrm{AW}}\mathrm{WP}P^{H}W^{H}{\tilde{H}}_{\mathrm{AW}}^{H}+\sigma_{W}^{2}I_{2M+1}\right), & H_{1}. \end{array}\right.$$

Let $R_{1}$ and $R_{0}$ denote the covariance matrices under $H_{1}$ and $H_{0}$, respectively, where $R_{1}≜\frac{P_{s}}{N}{\tilde{H}}_{\mathrm{AW}}\mathrm{WP}P^{H}W^{H}{\tilde{H}}_{\mathrm{AW}}^{H}+\sigma_{W}^{2}I_{2M+1}$ and $R_{0}≜\sigma_{W}^{2}I_{2M+1}$. The corresponding likelihood functions are expressed as

(21) $$P_{i}≜f\left(y_{W}|H_{i}\right)=\frac{1}{\pi^{2M+1}\det\left(R_{i}\right)}\exp\left(-y_{W}^{H}R_{i}^{-1}y_{W}\right)$$

where $i\in \left\{0,1\right\}$.

As shown in [14], when Willie employs the optimal detector, the minimum detection error probability (DEP) is given by

(22) $$\xi^{*}=1-V_{T}\left(P_{0},P_{1}\right)$$

where $V_{T}\left(P_{0},P_{1}\right)$ denotes the total variation (TV) distance, defined as

(23) $$V_{T}\left(P_{0},P_{1}\right)=\frac{1}{2}\int_{C^{2M+1}}\left|f\left(y_{W}|H_{0}\right)-f\left(y_{W}|H_{1}\right)\right|dy_{W}.$$

However, as indicated by (20) and (21), the received signals under both hypotheses follow high-dimensional complex Gaussian distributions, for which the TV distance is generally difficult to evaluate in a tractable closed form. To obtain a tractable sufficient condition for the DEP-based covertness requirement, Pinsker’s inequality is commonly adopted in covert communication to upper-bound the TV distance by the KL divergence [38]. Accordingly, the TV distance satisfies

(24) $$V_{T}\left(P_{0},P_{1}\right)⩽\sqrt{\frac{1}{2}D\left(P_{0}∥P_{1}\right)}$$

where $D\left(P_{0}∥P_{1}\right)$ denotes the KL divergence between the two distributions, given by

(25) $$\begin{array}{cc} D\;\left(P_{0}\;∥\;P_{1}\right)\; & =\int f\;\left(y_{W}|H_{0}\right)\ln\frac{f\;\left(y_{W}|H_{0}\right)}{f\;\left(y_{W}|H_{1}\right)}\;dy_{W}\\ \; & =\ln\det\;\left(I_{2M+1}+\frac{P_{s}}{N\sigma_{W}^{2}}{\tilde{H}}_{\mathrm{AW}}WPP^{H}W^{H}{\tilde{H}}_{\mathrm{AW}}^{H}\right)\\ \; & \;+\mathrm{tr}\;\left({\left(\frac{P_{s}}{N}{\tilde{H}}_{\mathrm{AW}}WPP^{H}W^{H}{\tilde{H}}_{\mathrm{AW}}^{H}+\sigma_{W}^{2}I_{2M+1}\right)}^{-1}\sigma_{W}^{2}I_{2M+1}\right)-\left(2M+1\right). \end{array}$$

Therefore, to satisfy the required covertness constraint $\xi^{*}⩾1-\varepsilon$, by combining (22) and (24), a more stringent constraint can be expressed as

(26) $$D\left(P_{0}∥P_{1}\right)⩽2\varepsilon^{2}.$$

### 3.2. KL Divergence Upper Bound Under Infinite-Harmonic Detection

When Willie performs infinite-harmonic joint observation and detection, the corresponding KL divergence can be expressed as the infinite-harmonic dimensional limit as

(27) $$\tilde{D}\left(P_{0}∥P_{1}\right)=\lim_{M\to \infty }D\left(P_{0}∥P_{1}\right).$$

Here, infinite-harmonic joint observation and detection refers to a worst-case detection model where Willie jointly exploits all harmonic components generated by time modulation, rather than only the fundamental component or a finite set of harmonics. As indicated by (25), under this worst-case scenario, (27) involves an infinite-dimensional covariance matrix, for which a closed-form expression is generally intractable and cannot be directly used for subsequent performance optimization or numerical evaluation. To address this issue, this subsection derives an analytically tractable upper bound on the KL divergence under infinite-harmonic joint observation, and provides the detailed derivation.

Further inspection of (27) reveals that the KL divergence upper bound consists of a log-determinant term and a trace term of an infinite-dimensional matrix. To facilitate tractable analysis, this subsection adopts an equivalent dimensionality-reduction approach, transforming (27) into a finite-dimensional matrix form. Specifically, the useful signal component observed by Willie is generated by only *K* user data streams, although the observation vector contains infinitely many harmonic components. Therefore, the covariance perturbation caused by Alice’s transmission has a low-rank structure determined by the number of users. By exploiting this structure, the infinite-dimensional determinant and trace terms can be transformed into expressions involving a *K*-dimensional effective correlation matrix. This transformation preserves the accumulated harmonic leakage effect while making the KL-divergence expression suitable for optimization.

**Lemma** **1.** *Let $A\in C^{n\times n},C\in C^{k\times k}$ be invertible matrices, and $U\in C^{n\times k}$ and $V\in C^{k\times n}$ be matrices of compatible dimensions, the following identity holds*

(28) $${\left(A+\mathrm{UCV}\right)}^{-1}=A^{-1}-A^{-1}U{\left(C^{-1}+VA^{-1}U\right)}^{-1}VA^{-1}.$$

For the KL divergence in (25), $\sqrt{\frac{P_{s}}{N}}{\tilde{H}}_{\mathrm{AW}}\mathrm{WP}\in C^{\left(2M+1\right)\times K}$, $\sqrt{\frac{P_{s}}{N}}P^{H}W^{H}{\tilde{H}}_{\mathrm{AW}}^{H}\in C^{K\times \left(2M+1\right)}$, and $\sigma_{W}^{2}I_{2M+1}$ is invertible matrices. Therefore, by invoking Lemma 1 and the linearity of the trace operator, (27) can be equivalently simplified as

(29) $$\begin{array}{cc} \; & \tilde{D}\left(P_{0}∥P_{1}\right)=\lim_{M\to \infty }\ln\det\left(I_{2M+1}+\frac{P_{s}}{N\sigma_{W}^{2}}{\tilde{H}}_{\mathrm{AW}}WPP^{H}W^{H}{\tilde{H}}_{\mathrm{AW}}^{H}\right)\\ \; & -\frac{P_{s}}{N\sigma_{W}^{2}}\mathrm{tr}\left[{\left(I_{K}+\frac{P_{s}}{N\sigma_{W}^{2}}P^{H}W^{H}{\tilde{H}}_{\mathrm{AW}}^{H}{\tilde{H}}_{\mathrm{AW}}WP\right)}^{-1}P^{H}W^{H}{\tilde{H}}_{\mathrm{AW}}^{H}{\tilde{H}}_{\mathrm{AW}}WP\right]. \end{array}$$

**Lemma** **2.** *Let $A\in C^{m\times n}$ and $B\in C^{n\times m}$ be matrices of compatible dimensions, it holds that*

(30) $$\det\left(I_{m}+\mathrm{AB}\right)=\det\left(I_{n}+\mathrm{BA}\right).$$

Therefore, by Lemma 2, the infinite-dimensional determinant term in (29) can be equivalently transformed into

(31) $$\det\left(I_{2M+1}+\frac{P_{s}}{N\sigma_{W}^{2}}{\tilde{H}}_{\mathrm{AW}}WPP^{H}W^{H}{\tilde{H}}_{\mathrm{AW}}^{H}\right)=\det\left(I_{K}+\frac{P_{s}}{N\sigma_{W}^{2}}P^{H}W^{H}{\tilde{H}}_{\mathrm{AW}}^{H}{\tilde{H}}_{\mathrm{AW}}WP\right).$$

By substituting (31) into (29), the KL divergence upper bound under the equivalent dimensionality reduction can be expressed as

(32) $$\begin{array}{cc} \tilde{D}\left(P_{0}∥P_{1}\right) & =\lim_{M\to \infty }\ln\det\left(I_{K}+\frac{P_{s}}{N\sigma_{W}^{2}}P^{H}W^{H}{\tilde{H}}_{\mathrm{AW}}^{H}{\tilde{H}}_{\mathrm{AW}}WP\right)\\ & \;-\frac{P_{s}}{N\sigma_{W}^{2}}\mathrm{tr}\left[{\left(I_{K}+\frac{P_{s}}{N\sigma_{W}^{2}}P^{H}W^{H}{\tilde{H}}_{\mathrm{AW}}^{H}{\tilde{H}}_{\mathrm{AW}}WP\right)}^{-1}P^{H}W^{H}{\tilde{H}}_{\mathrm{AW}}^{H}{\tilde{H}}_{\mathrm{AW}}WP\right]. \end{array}$$

It should be noted that the dimensionality-reduction procedure is based on exact matrix identities and does not introduce approximation or relaxation. Therefore, there is no tightness loss in this transformation; the conservative nature of the analysis mainly comes from the worst-case infinite-harmonic detection assumption at Willie.

After the above dimensionality reduction, the KL divergence reduces to the sum of a determinant term and a trace term of a *K*-dimensional matrix, thereby significantly reducing the computational complexity. However, the resulting expression still contains the non-closed-form term ${\tilde{H}}_{\mathrm{AW}}^{H}{\tilde{H}}_{\mathrm{AW}}$, which limits its direct application in optimization problems. To further obtain a tractable upper bound, let $C_{\mathrm{AW}}≜{\tilde{H}}_{\mathrm{AW}}^{H}{\tilde{H}}_{\mathrm{AW}}$ be defined accordingly, which represents the equivalent channel correlation matrix at Willie, characterizing the aggregated spatial–harmonic coupling effect induced by time modulation and antenna array responses. To make this aggregated spatial–harmonic coupling effect analytically tractable, a free-space path-loss model is adopted in this paper for the Alice–Willie channel. Under a free-space path-loss model, the channel from the *n*-th antenna element to Willie at the *m*-th harmonic is given by

(33) $$h_{\mathrm{AW},n,m}=\sqrt{\frac{g_{0}}{d_{W}^{\nu }}}e^{-j2\pi \left(n-1\right)\frac{d}{\lambda }\sin\left(\theta_{W}\right)}e^{-j2\pi mf_{P}\tau }$$

where τ=dWdWcc denotes the propagation delay, $d_{W}$ and $\theta_{W}$ represent the distance and AoD from Alice to Willie, respectively. By combining with (33), the expression of $C_{\mathrm{AW}}$ can be rewritten as

(34) $$\begin{array}{cc} C_{\mathrm{AW}}\; & \;=\lim_{M\to \infty }{\left(\sum_{i=1}^{N}H_{\mathrm{AW},i}A_{i}\right)}^{H}\left(\sum_{j=1}^{N}H_{\mathrm{AW},j}A_{j}\right)\\ \; & \;=\frac{g_{0}}{d_{W}^{\nu }}\sum_{i=1}^{N}\sum_{j=1}^{N}e^{j2\pi \left(i-j\right)\frac{d}{\lambda }\sin\left(\theta_{W}\right)}e^{j(\varphi_{j}-\varphi_{i})}\lim_{M\to \infty }A_{i}^{H}A_{j}. \end{array}$$

Hence, the problem reduces to obtaining a closed-form characterization of each summation term in $\lim_{M\to \infty }A_{i}^{H}A_{j}$, which characterizes the asymptotic harmonic coupling induced by time modulation between antenna elements *i* and *j* in the frequency domain. Intuitively, $A_{i}^{H}A_{j}$ measures the correlation between the harmonic spectra generated by the time-modulation sequences of antenna elements *i* and *j*. Taking the limit M→∞ accumulates the contribution of all harmonic orders, thereby capturing the full harmonic leakage effect.

**Theorem** **1.** *Under the infinite-harmonic joint observation and detection at Willie, the expression of $\lim_{M\to \infty }A_{i}^{H}A_{j}$ can be reformulated based on (17) as*

(35) $$\begin{array}{c} \lim_{M\to \infty }A_{i}^{H}A_{j}=\lim_{M\to \infty }\left[\begin{array}{ccc} \sum_{m=-M+Q}^{M+Q}\alpha_{i,m}^{*}\alpha_{j,m} & \cdots & \sum_{m=-M+Q}^{M+Q}\alpha_{i,m}^{*}\alpha_{j,m-2Q}\\ \vdots & \ddots & \vdots \\ \sum_{m=-M-Q}^{M-Q}\alpha_{i,m}^{*}\alpha_{j,m+2Q} & \cdots & \sum_{m=-M-Q}^{M-Q}\alpha_{i,m}^{*}\alpha_{j,m} \end{array}\right] \end{array}$$

*where the closed-form expressions of the diagonal and off-diagonal elements of $\lim_{M\to \infty }A_{i}^{H}A_{j}$ are given in (A8) and (A20), respectively.*

**Proof.**  The proof is provided in Appendix A. □

It can be observed that each entry of $\lim_{M\to \infty }A_{i}^{H}A_{j}$ can be expressed as an infinite series in terms of the harmonic coefficients, which are determined by the time-modulation-induced weighting factors. Since the time-modulation parameters satisfy $\tau_{n},{\bar{\tau }}_{n}\in \left[0,1\right]$, the corresponding harmonic coefficients decay monotonically with respect to the harmonic order, ensuring the existence of the limit.

## 4. Max–Min Rate Design Under Strict Covert Constraints

This section investigates the optimization problem of maximizing the minimum covert transmission rate under the upper-bounded KL divergence constraint. A genetic-algorithm-based joint optimization framework is developed to enable the coordinated design of the time-modulation sequence, power allocation, and spatial phase parameters.

### 4.1. Optimization Problem Formulation

This paper focuses on improving the transmission rate of the legitimate Alice–Bob link while ensuring reliable covert communication under a given covertness constraint. It is assumed that Alice transmits the legitimate signals with a total power $P_{s}$, which is allocated among different data streams according to predefined power allocation coefficients $\rho_{k}$. Based on (11), the signal-to-interference-plus-noise ratio (SINR) at the *k*-th Bob can be expressed as

(36) $$\mathrm{SIN}R_{k}=\frac{\rho_{k}P_{s}{\left|h_{\mathrm{AB},k}^{T}\Phi Bw_{k}\right|}^{2}}{\sum_{j\neq k}\rho_{j}P_{s}{\left|h_{\mathrm{AB},k}^{T}\Phi Bw_{j}\right|}^{2}+N\sigma_{k}^{2}}.$$

Therefore, the covert transmission rate of the *k*-th Bob can be further given by

(37) $$R_{k}=\mathrm{lo}g_{2}\left(1+\mathrm{SIN}R_{k}\right).$$

In covert communication systems, the performance of the legitimate link is not only determined by the average rate but is also constrained by the worst-case user. To guarantee a satisfactory quality-of-service (QoS) for all legitimate users under the covertness constraint, a max–min fairness criterion is adopted. Specifically, the minimum covert transmission rate among all users is maximized to ensure fair rate allocation in the multi-user scenario. Accordingly, the covert transmission rate optimization problem for the Alice–Bob links is formulated as

(38) $$\begin{array}{cc} P1:\;\; & \max_{\rho ,\tau ,\bar{\tau },\varphi }\;\min_{k\in K}R_{k}\\ s.t.\;\; & \tilde{D}\left(P_{0}∥P_{1}\right)\leq 2\varepsilon^{2},\\ \; & \rho_{k}\geq 0,\;\sum_{k\in K}\rho_{k}=1,\;\forall k\in K,\\ \; & 0_{N\times 1}≼\tau ≼1_{N\times 1},\\ \; & 0_{N\times 1}≼\bar{\tau }≼1_{N\times 1},\\ \; & \varphi_{n}\in \left(0,2\pi \right],\;\forall n\in N \end{array}$$

where $\tilde{D}\left(P_{0}∥P_{1}\right)$ denotes the covertness constraint function, and ε represents the covertness parameter, which determines the detection capability of Willie. The vectors τ and $\bar{\tau }$ denote the time-modulation sequence design parameters. The vector $\rho ≜[\rho_{1},\rho_{2},\cdots ,\rho_{K}]$ represents the power allocation ratios among different data streams at Alice. The vector $\varphi ≜[\varphi_{1},\varphi_{2},\cdots ,\varphi_{N}]$ denotes the phase-shift design parameters of the phase shifters at the transmitter.

### 4.2. Genetic Algorithm-Based Maximization of the Minimum Transmission Rate

The optimization problem P1 under consideration is a non-convex joint design problem with a max–min objective, subject to a nonlinear covertness constraint derived from the KL divergence upper bound, as well as coupled variables including power allocation, time-modulation parameters, and spatial phase control. Moreover, as shown in Section 3.2, the covertness constraint involves an infinite number of harmonic components, resulting in a highly irregular and non-convex feasible region. These characteristics make conventional convex optimization methods or gradient-based iterative algorithms inapplicable and prone to being trapped in local optima. Therefore, a GA-based approach is adopted as a derivative-free heuristic solver to search for a high-quality feasible solution to problem P1.

To provide a concise characterization of the computational burden, the complexity of the proposed GA-based algorithm is analyzed in terms of the chromosome dimension, the number of fitness evaluations, and the cost of each fitness evaluation. Let $N_{\mathrm{pop}}$ denote the population size and $G_{\max}$ denote the maximum number of generations. For each individual, the chromosome consists of ρ, τ, $\bar{\tau }$, and φ. Therefore, the chromosome dimension is D=K+3N. For each candidate solution, the complexity of one fitness evaluation is $O\left(N^{2}K+NK^{2}+K^{3}\right)$. Since the GA evaluates approximately $N_{\mathrm{pop}}G_{\max}$ candidate solutions during the optimization process, the overall computational complexity of the proposed GA-based algorithm is given by $O\left[N_{\mathrm{pop}}G_{\max}\left(K+3N+N^{2}K+NK^{2}+K^{3}\right)\right]$.

## 5. Numerical Results

This section presents numerical simulation results to validate the performance of the proposed TMA-SDMA covert communication system. The inter-element spacing of the TMA is set to d=λ/2. It is assumed that the noise power at the Bobs and Willie is identical, i.e., $\sigma^{2}=\sigma_{k}^{2}=\sigma_{W}^{2}$. The detailed simulation parameters are summarized in Table 1.

### 5.1. Impact of Detectable Harmonic Order at Willie on Covertness

**Figure 3:**
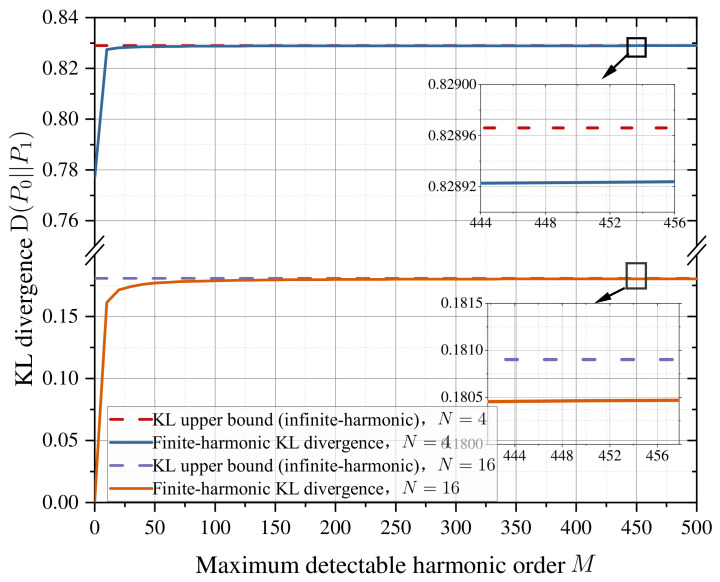
KL divergence versus Willie’s maximum detectable harmonic order.

To illustrate the impact of the maximum detectable harmonic order at Willie on system covertness, Figure 3 depicts the variation of the KL divergence under different *M*. For simplicity, a one-by-one time-modulation sequence is adopted at the TMA transmitter, and all phase shifters are set to π. It can be observed that the KL divergence grows rapidly at small harmonic orders and gradually saturates as *M* becomes large. This trend indicates that the accumulated leakage observed by Willie is bounded, because the power of high-order TMA-induced harmonics gradually attenuates in the frequency domain. Moreover, for N=4 and 16, when M≈450, the gap between the finite-harmonic and infinite-harmonic results remains within $10^{-4}$, validating the accuracy of the derived closed-form upper bound. In addition, as *M* increases from 0 to 450, the KL divergence under both N=4 and 16 increases by approximately 0.06 and 0.18, respectively. The result confirms that considering only low-order harmonics may underestimate Willie’s detection capability, while the infinite-harmonic model provides a more reliable characterization of covert performance.

**Figure 4:**
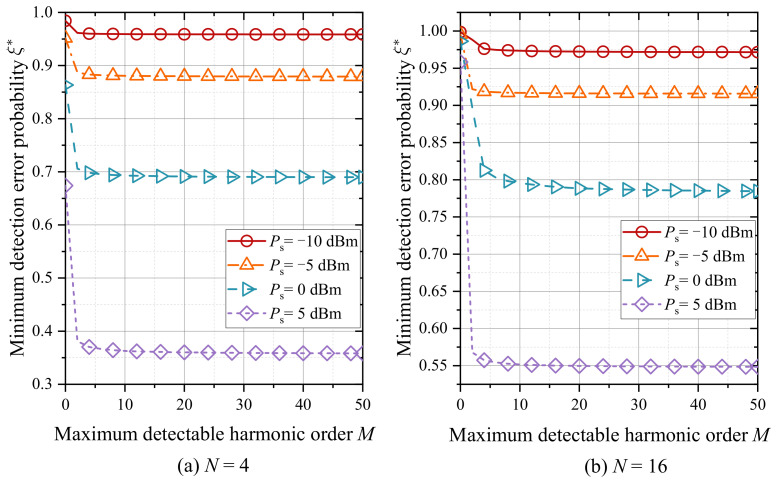
Minimum detection error probability versus Willie’s maximum detectable harmonic order.

To further evaluate the impact of Willie’s maximum detectable harmonic order on the binary detection performance, Figure 4 shows the impact of Willie’s maximum detectable harmonic order *M* on the minimum detection error probability $\xi^{*}$ under different transmit power $P_{s}$. Specifically, Figure 4a corresponds to the small-scale setting with N=4, while Figure 4b considers a larger array with N=16. As shown in Figure 4a,b, $\xi^{*}$ decreases monotonically as *M* increases across all transmit power settings. This is because a larger detectable harmonic order provides Willie with richer frequency-domain observations for joint detection. A further comparison across different $P_{s}$ reveals that, as $P_{s}$ increases from −10 dBm to 5 dBm, the minimum detection error probability $\xi^{*}$ decreases from 0.95 to 0.36 for N=4, and from 0.97 to 0.55 for N=16. This indicates that higher transmit power makes multi-harmonic leakage more detectable by Willie. Meanwhile, compared with the case of N=4, the system with N=16 still maintains a higher detection error probability, demonstrating that increasing the number of antenna elements helps improve the system covertness performance. Moreover, $\xi^{*}$ becomes more sensitive to *M* at higher $P_{s}$, while remaining relatively insensitive at lower power levels. Therefore, multi-harmonic joint detection becomes particularly critical when the system operates with a relatively high transmit power.

### 5.2. The Convergence Behavior of the Algorithms Proposed

**Figure 5:**
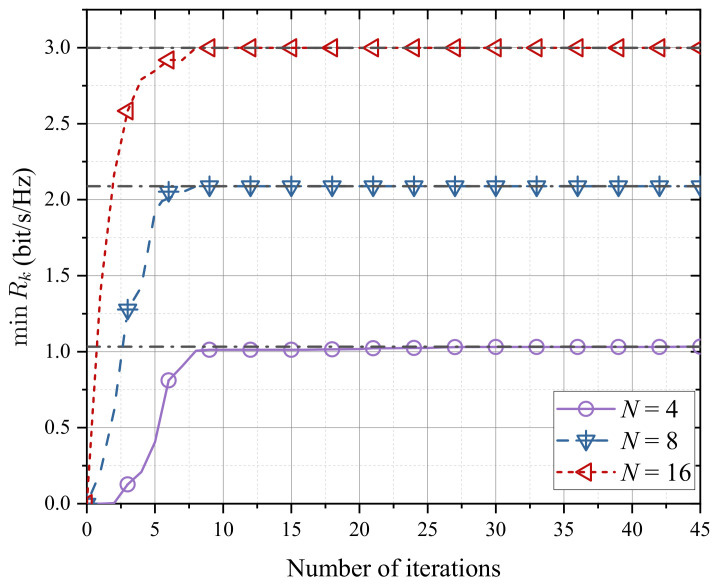
Convergence behavior of the minimum covert transmission rate for different numbers of antenna elements.

Figure 5 presents the convergence behavior of the proposed algorithm under different numbers of antenna elements. The observations are detailed as follows. For all considered cases, the minimum covert transmission rate increases rapidly during the initial iterations and then gradually converges to a stable value. This indicates that the proposed algorithm can effectively improve the objective value within a limited number of iterations. Specifically, when N=4, the minimum covert transmission rate converges to approximately 1.0 bit/s/Hz after about 10 iterations. When the number of antenna elements increases to N=8 and N=16, the proposed algorithm still maintains stable convergence behavior and achieves higher minimum covert transmission rates, reaching approximately 2.1 bit/s/Hz and 3.0 bit/s/Hz, respectively. This result indicates that increasing the number of antenna elements does not prevent the proposed algorithm from converging. Instead, a larger antenna array provides more spatial degrees of freedom, which helps improve multi-user interference suppression and covert beamforming design. Therefore, the proposed scheme can benefit from an increased number of antenna elements while maintaining stable convergence performance.

### 5.3. Covertness Performance Comparison Under Different Constraints

Building on the above analysis of the detectable harmonic order at Willie, we further evaluate the performance of different covertness-constrained optimization schemes. In the subsequent simulations, the time modulation sequence, power allocation and spatial phase are jointly optimized under various covertness constraints, and the resulting covert transmission rate as well as the corresponding KL divergence are compared. Specifically, two system structures are considered, namely the proposed TMA-SDMA system with phase shifters (denoted as TMA+PS) and the conventional TMA structure (denoted as TMA [33]). For each structure, three optimization strategies are adopted:

- no covertness constraint: Without covertness constraint;
- fund.-KL Opt.: KL divergence constraint based on fundamental-harmonic detection;
- UB-KL Opt.: KL divergence upper-bound constraint based on infinite-harmonic joint detection.

**Figure 6:**
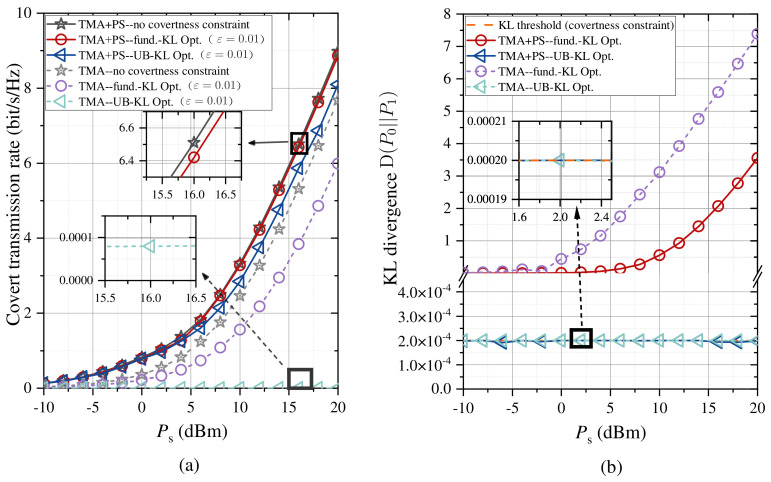
Relationship between covert transmission rate, KL divergence, and total transmit power: (**a**) covert transmission rate versus total transmit power; (**b**) KL divergence versus total transmit power. The “TMA” scheme is adopted from [33].

To evaluate the impact of different covertness-constrained optimization schemes on the performance of the TMA-SDMA covert communication system, Figure 6 presents the covert transmission rate and the corresponding KL divergence under different transmit power $P_{s}$. Specifically, Figure 6a shows the covert transmission rate versus $P_{s}$, while Figure 6b depicts the corresponding KL divergence when Willie performs infinite harmonic joint detection. Obviously, the proposed “TMA+PS–UB-KL Opt.” scheme achieves a monotonically increasing covert transmission rate as $P_{s}$ increases. Meanwhile, the corresponding KL divergence remains strictly below the “KL threshold” across the entire power range and is nearly insensitive to $P_{s}$. This demonstrates that the “TMA+PS–UB-KL Opt.” design can effectively improve the transmission rate while rigorously satisfying the covertness requirement. Under the same constraint, the “TMA [33]–UB-KL Opt.” scheme yields an almost zero covert transmission rate when the covertness constraint is enforced. This performance gap indicates that the proposed transmitter structure, by introducing additional spatial phase control, provides enhanced beamforming flexibility. Furthermore, both the “TMA [33]–fund.-KL Opt.” scheme and the “TMA+PS–fund.-KL Opt.” scheme achieve higher covert transmission rates in the medium-to-high power regime, approaching the rate upper bound without covertness constraints. However, as shown in Figure 6b, when Willie performs infinite harmonic joint detection, the actual KL divergence increases rapidly with $P_{s}$ and significantly exceeds the prescribed threshold in the high-power regime. Hence, the apparent rate gain obtained under the fundamental-harmonic-based constraint is achieved at the cost of violating the actual covertness requirement. These results highlight that, a covertness constraint based only on the fundamental harmonic may overestimate the achievable covert performance in TMA-SDMA systems. In contrast, the proposed UB-KL-based optimization provides a more reliable design criterion by accounting for the accumulated leakage from infinite harmonic components.

**Figure 7:**
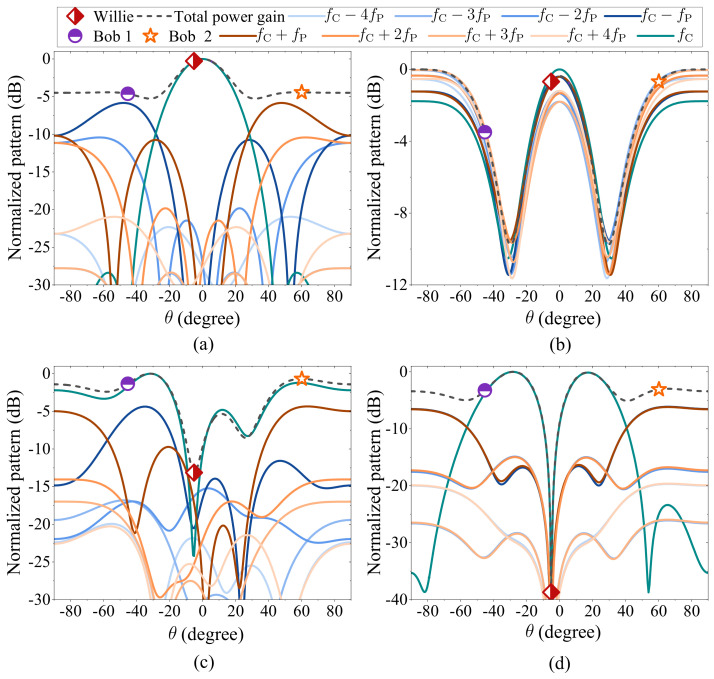
Comparison of normalized beampatterns under different optimization schemes: (**a**) TMA [33], fund.–KL Opt. (**b**) TMA [33], UB–KL Opt. (**c**) TMA+PS, fund.–KL Opt. (**d**) TMA+PS, UB–KL Opt.

Figure 7 illustrates the normalized harmonic radiation patterns under different covertness-constrained optimization schemes with $P_{s}=12$ dBm. For clarity, only the patterns corresponding to harmonic orders from −4 to +4 are shown. Overall, as depicted in Figure 7a,b, the “TMA [33]” scheme, exhibits significantly weaker harmonic suppression in the direction of Willie compared with the proposed “TMA+PS” scheme shown in Figure 7c,d. This is because the proposed “TMA+PS” transmitter incorporates phase shifters into the antenna structure, thereby enabling enhanced phase control and providing additional spatial degrees of freedom. As a result, all harmonic beams achieve improved beam alignment toward legitimate users while offering stronger suppression toward Willie. Furthermore, as shown in Figure 7c,d, the proposed “TMA+PS–UB-KL Opt.” system achieves more pronounced harmonic suppression in the direction of Willie. Specifically, while the fundamental component is adaptively aligned with the legitimate users, all harmonic components form deep nulls toward Willie. By comparison, under the “TMA+PS–fund.-KL Opt.” scheme, only the fundamental component is explicitly controlled, while higher-order harmonics remain insufficiently suppressed, leading to increased total power gain in Willie’s direction. These observations further indicate that when Willie performs detection over non-carrier frequency bands, infinite harmonic modeling is essential for accurately characterizing the detection capability of Willie and achieving reliable covert communication in TMA-SDMA systems.

**Figure 8:**
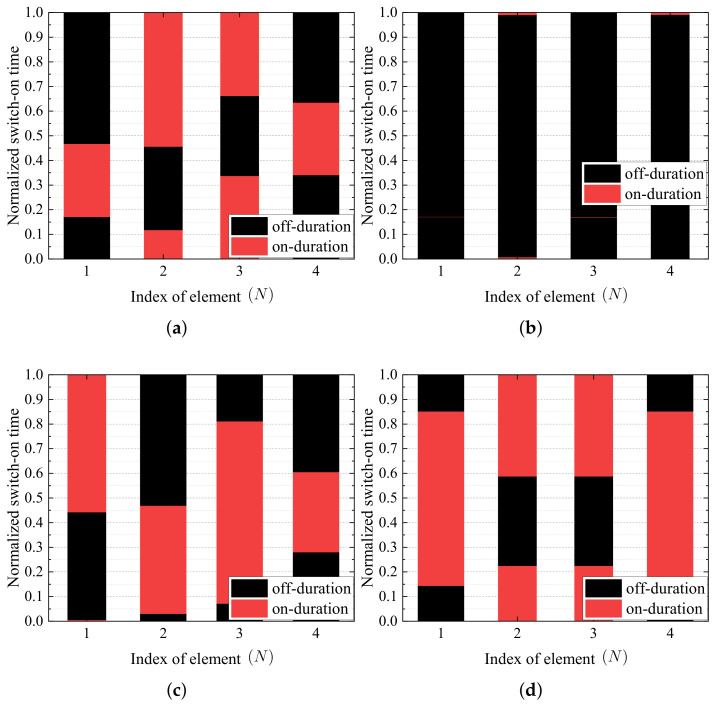
Comparison of normalized switch-on time under different optimization schemes: (**a**) TMA [33], fund.–KL Opt. (**b**) TMA [33], UB–KL Opt. (**c**) TMA+PS, fund.–KL Opt. (**d**) TMA+PS, UB–KL Opt.

Figure 8 compares the optimized normalized switch-on time of all antenna elements under different schemes to demonstrate the effectiveness of time modulation. It can be observed that the optimized time-modulation sequences differ significantly among different schemes. This is because the normalized duty cycle directly affects the magnitude of the harmonic coefficients, while the normalized switching midpoint affects phase distribution. Therefore, different time-modulation sequences lead to different harmonic energy distributions and beamforming characteristics. For the “TMA [33]” scheme, the optimized switch-on durations are mainly adjusted under limited spatial-domain control capability to satisfy the corresponding KL-divergence constraint. In contrast, the “TMA+PS” scheme provides additional phase-shift degrees of freedom, enabling joint optimization of the time-modulation sequence and spatial phase control. As a result, the optimized switch-on time become more flexible across antenna elements.

### 5.4. Impact of System Parameters on Covert Communication Performance

**Figure 9:**
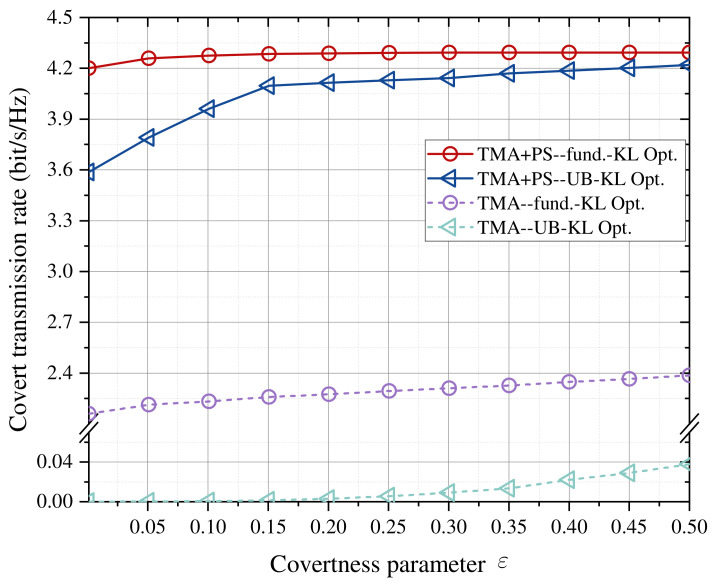
Covert transmission rate versus covertness parameter under different schemes. The “TMA” scheme is adopted from [33].

To investigate the performance advantage of the proposed TMA-SDMA covert communication system under different covertness parameters, Figure 9 compares the covert transmission rates of different schemes under varying covertness parameter ε, with $P_{s}=12$ dBm. The results show that the “TMA+PS” system consistently outperforms the conventional “TMA [33]” scheme over the entire range of ε. Specifically, within ε∈[0.1,0.5], the “TMA+PS–fund.-KL Opt.” scheme achieves a stable covert transmission rate of approximately 4.2 bit/s/Hz, whereas the “TMA [33]–fund.-KL Opt.” scheme only attains about 2.2–2.4 bit/s/Hz. This performance gap confirms that the additional spatial phase control in the proposed TMA+PS structure provides more effective beamforming flexibility. In contrast, when the covertness constraint is based on the KL-divergence upper bound, the achievable covert transmission rate of the conventional “TMA [33]” scheme remains close to zero over the entire range of ε, indicating that the conventional TMA structure has limited ability to satisfy the stricter infinite-harmonic covertness constraint. Further examining the impact of different constraint formulations, the proposed “TMA+PS–UB-KL Opt.” scheme achieves a covert transmission rate of approximately 3.6 bit/s/Hz in the stringent covertness regime (small ε), which approaches 4.2 bit/s/Hz as ε becomes larger. Consequently, the performance gap between the “TMA+PS–UB-KL Opt.” scheme and the “TMA+PS–fund.-KL Opt.” scheme diminishes and becomes negligible in the relaxed covertness regime. The proposed “TMA+PS–UB-KL Opt.” scheme is still capable of achieving high covert transmission rates even under strict covertness requirements. Overall, the proposed phase-enhanced TMA-SDMA design provides a better trade-off between covertness and transmission performance.

**Figure 10:**
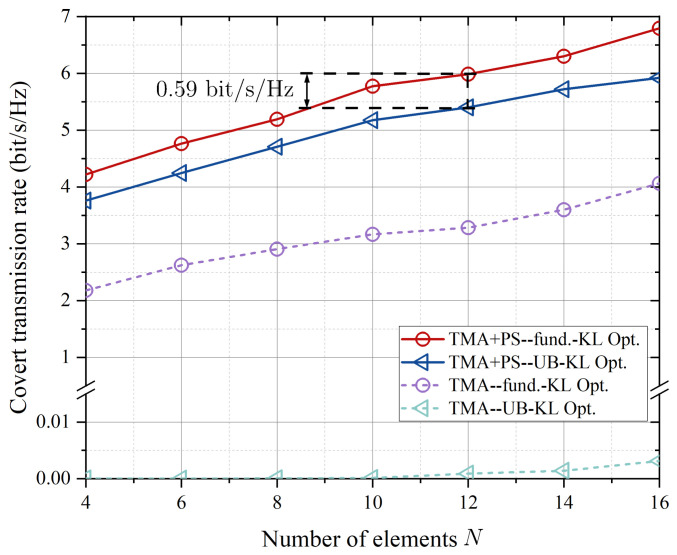
Covert transmission rate versus number of array elements under different schemes. The “TMA” scheme is adopted from [33].

To evaluate the scalability of the proposed TMA-SDMA covert communication system with increasing spatial dimensions, Figure 10 illustrates the covert transmission rate versus the number of array elements *N* under different optimization schemes, with $P_{s}=12$ dBm. A clear trend is that the covert transmission rates of all schemes increase steadily as *N* grows. This is attributed to the higher array gain and richer spatial degrees of freedom provided by larger antenna arrays, which enhance beam focusing toward Bobs and improve the covert transmission rate. Further comparison across different covertness constraints reveals that, for the proposed “TMA+PS” system, the “TMA+PS–UB-KL Opt.” scheme continues to achieve increasing covert transmission rates with *N* while strictly satisfying the infinite-harmonic covertness constraint. The performance gap between the “TMA+PS–UB-KL Opt.” scheme and the “TMA+PS–fund.-KL Opt.” scheme remains approximately 0.59 bit/s/Hz, indicating that the stricter infinite-harmonic constraint only causes a moderate rate loss for the proposed TMA+PS structure. In contrast, for the conventional TMA [33]” structure, extending the covertness constraint from the fundamental-harmonic model to the infinite-harmonic joint detection model leads to a significant degradation in the achievable covert transmission rate, although the TMA [33]–fund.-KL Opt.” scheme also benefits from increasing *N*. Therefore, the proposed “TMA+PS–UB-KL Opt.” framework can more effectively exploit additional spatial degrees of freedom under stringent covertness requirements, demonstrating better scalability with respect to the number of array elements. This result is also consistent with the use of ZF precoding in the considered system. A larger number of array elements provides richer spatial degrees of freedom, which is beneficial for multi-user interference suppression and therefore improves the achievable covert transmission performance.

### 5.5. Impact of Practical Channel Effects on Covert Transmission

**Figure 11:**
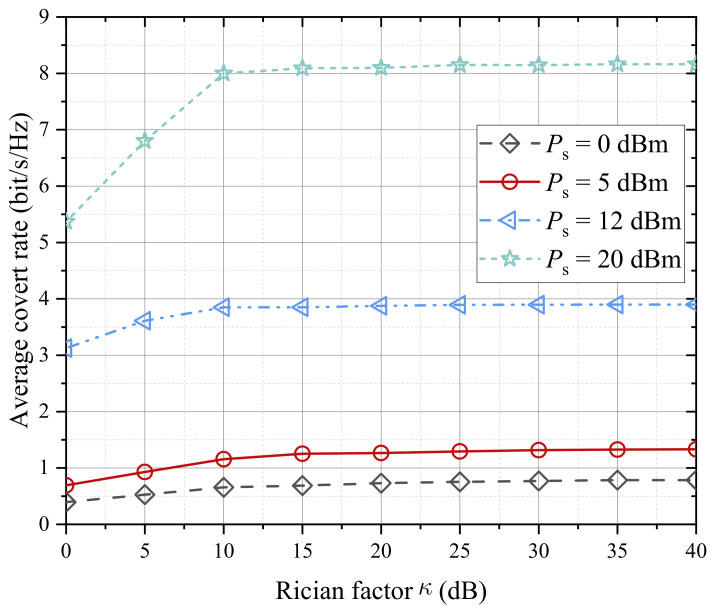
Covert transmission rate versus the Rician factor under different transmit powers.

To examine the robustness of the proposed scheme in the presence of NLoS scattering, we further evaluate its performance over a Rician fading channel in this section. The channel $h_{\mathrm{AB},k}$ and $H_{\mathrm{AW},n}$ are modeled as $h_{\mathrm{AB},k}^{\prime }=\sqrt{\frac{\kappa }{\kappa +1}}h_{\mathrm{AB},k}+\sqrt{\frac{1}{\kappa +1}}\chi_{k}$ and $h_{\mathrm{AW},n,m}^{\prime }=\sqrt{\frac{\kappa }{\kappa +1}}h_{\mathrm{AW},n,m}+\sqrt{\frac{1}{\kappa +1}}\chi_{W,n,m}$ as in [39], where κ denotes the Rician factor, $\chi_{k}∼CN\left(0,1\right)$ and $\chi_{W,n,m}∼CN\left(0,1\right)$, respectively. The channel approaches the LoS case as κ→∞, while it reduces to the Rayleigh fading case when κ=0. Figure 11 shows the average covert transmission rate versus the Rician factor under different transmit powers. It can be observed that the average covert transmission rate increases as the Rician factor grows and then gradually saturates. This is because a larger Rician factor corresponds to a stronger LoS component and a more stable spatial channel structure, which improves the effectiveness of the proposed beamforming and harmonic-domain optimization. By contrast, when the Rician factor is small, the NLoS scattered component becomes more significant, resulting in stronger channel fluctuations and lower covert transmission rates. Moreover, a higher transmit power leads to a higher average covert rate under the same Rician factor. The results demonstrate that the proposed framework remains effective under Rician fading channels and is not limited to the deterministic free-space channel model.

**Figure 12:**
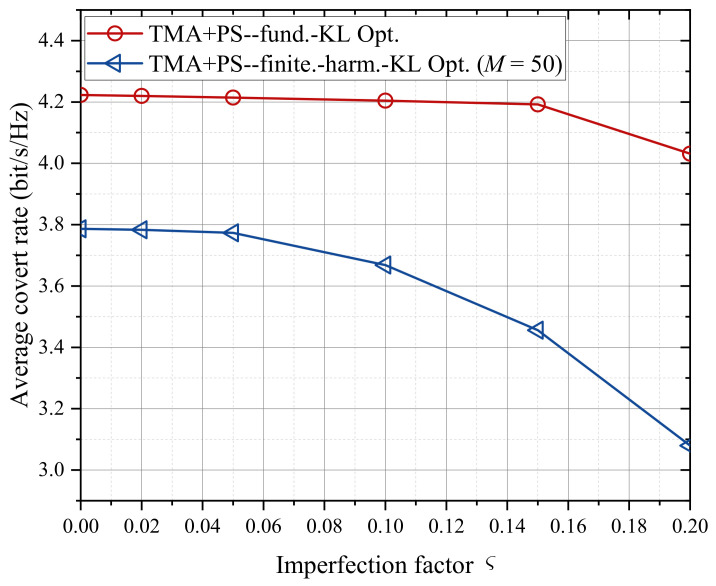
Covert transmission rate versus the imperfection factor under different covertness constraints.

To further evaluate the robustness of the proposed scheme in practical wireless environments, we consider the covert transmission performance under imperfect Alice–Willie CSI. In practical TMA-SDMA covert communication systems, the Alice–Willie channel response used for beamforming design and covertness analysis is usually constructed based on estimated propagation parameters, including Willie’s angular information. Estimation errors in these parameters may cause the reconstructed Alice–Willie channel response to deviate from the actual one, leading to a mismatch between the estimated and actual Alice–Willie CSI. Therefore, we further investigate the system performance under imperfect Alice–Willie CSI. Following the model in [40], the imperfect CSI is represented as

(39) $$H_{\mathrm{AW},n}={\hat{H}}_{\mathrm{AW},n}\;+\;\Delta H_{\mathrm{AW},n}$$

where ${\hat{H}}_{\mathrm{AW},n}$ denotes the estimated channel between the *n*-th antenna element of Alice and Willie, and $\Delta H_{\mathrm{AW},n}$ represents the channel estimation error. The channel uncertainty is assumed to satisfy $∥\Delta H_{\mathrm{AW},n}∥⩽\varsigma ∥{\hat{H}}_{\mathrm{AW},n}∥$, where ς characterizes the level of CSI uncertainty. Figure 12 illustrates the average covert transmission rate under different levels of CSI uncertainty. It should be noted that, under imperfect CSI, the covariance matrix $C_{\mathrm{AW}}$ involved in the KL-divergence analysis at Willie is jointly affected by both the channel uncertainty term and the harmonic coupling effect introduced by TMA. As a result, deriving a closed-form upper bound on the KL divergence under the infinite-harmonic joint-detection scenario becomes analytically intractable. Therefore, a finite-harmonic joint-detection model is adopted as a numerical approximation, where the maximum detectable harmonic order *M* is set to 50. As observed from Figure 12, the average covert transmission rate under both detection constraints gradually decreases as the CSI imperfection factor ς increases. This demonstrates that the proposed scheme exhibits robustness to CSI mismatch; however, its performance gradually deteriorates as the CSI uncertainty increases. The primary reason is that the beamforming vectors designed based on the estimated CSI deviate from the actual Alice–Willie channel, thereby weakening the spatial suppression capability against covert signal leakage. Meanwhile, the harmonic energy distribution generated by the TMA may deviate from its intended design due to CSI mismatch, leading to increased leakage of certain frequency-domain harmonic components and, consequently, improved detection capability at Willie.

## 6. Conclusions

This paper investigated a TMA-based multi-user SDMA covert communication system under the infinite-harmonic assumption. The analysis revealed the non-negligible role of high-order harmonic components in covert transmission. High-order harmonics can introduce signal leakage toward non-target directions and affect the warden’s detection performance. Consequently, finite-harmonic models may lead to an overly optimistic evaluation of covertness in practical TMA-based systems. To enable tractable performance characterization, an upper bound on the KL divergence under infinite harmonics was derived and used for covert communication design. Based on this analytical framework, a max–min optimization problem was formulated to improve the minimum user rate under the covertness constraint. A GA-based algorithm was developed to jointly optimize the time-modulation sequence, power allocation, and spatial phase parameters. Numerical results showed that the proposed design improves covert transmission performance while suppressing signal leakage toward the warden. Overall, the results demonstrate the importance of infinite-harmonic-aware modeling and optimization for reliable high-covertness multi-user TMA-SDMA system design.

## Figures and Tables

**Table 1:** Simulation parameters.

Parameter	Notation	Value
Number of Bobs	*K*	2
Distance of Bobs	$\left\{d_{1},d_{2}\right\}$	{2750,2500} m
Direction of Bobs	$\left\{\theta_{1},\theta_{2}\right\}$	$\left\{60^{\circ },-45^{\circ }\right\}$
Distance of Willie	$d_{W}$	800 m
Direction of Willie	$\theta_{W}$	$-5^{\circ }$
Covertness parameter	ε	0.01
Center frequency	$f_{c}$	2.4 GHz
Number of TMA elements	*N*	4
Harmonic order of precoding	*Q*	2
Signal bandwidth	*B*	8 MHz
Noise power	$\sigma^{2}$	−105 dBm
Path loss exponent	ν	2
Reference path loss	$g_{0}$	−40 dB

## Data Availability

The original contributions presented in this study are included in the article. Further inquiries can be directed to the corresponding author.

## Abbreviations

The following abbreviations are used in this manuscript:

TMA	Time-Modulated Arrays
RF	Radio Frequency
GA	Genetic Algorithm
SDMA	Space-Division Multiple Access
AWGN	Additive White Gaussian Noise
DUC	Digital Up-Converter
DAC	Digital-to-Analog Converter
AoD	Aangle of Departure
SINR	Signal-to-Interference-plus-Noise Ratio

## Appendix A. Closed-Form Expressions of $\lim_{M\to \infty }A_{i}^{H}A_{j}$

We provide here the detailed derivation of the closed-form expressions for the entries of $\lim_{M\to \infty }A_{i}^{H}A_{j}$, distinguishing the diagonal and off-diagonal cases. In the case of q=0, the diagonal entries of $\lim_{M\to \infty }A_{i}^{H}A_{j}$ can be expressed by combining (6) as

(A1) $$\begin{array}{cc} \sum_{m=-\infty }^{\infty }\alpha_{i,m}^{*}\alpha_{j,m}\; & =\sum_{m=-\infty }^{\infty }\tau_{i}\mathrm{sinc}\left(\pi m\tau_{i}\right)\;\tau_{j}\mathrm{sinc}\left(\pi m\tau_{j}\right)\;e^{j2\pi m({\bar{\tau }}_{i}-{\bar{\tau }}_{j})}\\ \; & =\underset{\left(a\right)}{\underset{︸}{\sum_{\begin{array}{c} m=-\infty \\ m\neq 0 \end{array}}^{\infty }\frac{\sin\left(\pi m\tau_{i}\right)\sin\left(\pi m\tau_{j}\right)}{\pi^{2}m^{2}}e^{j2\pi m({\bar{\tau }}_{i}-{\bar{\tau }}_{j})}}}+\tau_{i}\tau_{j}. \end{array}$$

To simplify the non-zero term $\left(a\right)$ in (A1), trigonometric identities and the even symmetry property of the cosine function are utilized, allowing $\left(a\right)$ to be further rewritten as

(A2) $$\begin{array}{cc} \left(a\right)\; & =\sum_{\begin{array}{c} m=-\infty \\ m\neq 0 \end{array}}^{\infty }\frac{\cos\;\left(\pi m(\tau_{i}-\tau_{j})\right)-\cos\;\left(\pi m(\tau_{i}+\tau_{j})\right)}{2\pi^{2}m^{2}}e^{j2\pi m({\bar{\tau }}_{i}-{\bar{\tau }}_{j})}\\ \; & =\frac{1}{2\pi^{2}}(\sum_{m=1}^{\infty }\frac{\cos\;\left(\pi m(\tau_{i}-\tau_{j}+2\left({\bar{\tau }}_{i}-{\bar{\tau }}_{j}\right))\right)}{m^{2}}+\sum_{m=1}^{\infty }\frac{\cos\;\left(\pi m(\tau_{i}-\tau_{j}-2\left({\bar{\tau }}_{i}-{\bar{\tau }}_{j}\right))\right)}{m^{2}}\\ \; & \;-\sum_{m=1}^{\infty }\frac{\cos\;\left(\pi m(\tau_{i}+\tau_{j}+2\left({\bar{\tau }}_{i}-{\bar{\tau }}_{j}\right))\right)}{m^{2}}-\sum_{m=1}^{\infty }\frac{\cos\;\left(\pi m(\tau_{i}+\tau_{j}-2\left({\bar{\tau }}_{i}-{\bar{\tau }}_{j}\right))\right)}{m^{2}}). \end{array}$$

Since $\tau_{n},{\bar{\tau }}_{n}\in \left[0,1\right]$, auxiliary variables $x_{1},x_{2},x_{3},x_{4}$ are introduced as

(A3) $$\left\{\begin{array}{cc} x_{1} & ≜\tau_{i}-\tau_{j}+2\left({\bar{\tau }}_{i}-{\bar{\tau }}_{j}\right),\;x_{1}\in \left[-3,3\right],\\ x_{2} & ≜\tau_{i}-\tau_{j}-2\left({\bar{\tau }}_{i}-{\bar{\tau }}_{j}\right),\;x_{2}\in \left[-3,3\right],\\ x_{3} & ≜\tau_{i}+\tau_{j}+2\left({\bar{\tau }}_{i}-{\bar{\tau }}_{j}\right),\;x_{3}\in \left[-2,4\right],\\ x_{4} & ≜\tau_{i}+\tau_{j}-2\left({\bar{\tau }}_{i}-{\bar{\tau }}_{j}\right),\;x_{4}\in \left[-2,4\right]. \end{array}\right.$$

**Lemma** **A1.**
*For a variable x satisfying −2π⩽x⩽2π, the following holds*

(A4)
$$\sum_{r=1}^{\infty }\frac{\cos\left(r\left|x\right|\right)}{r^{2}}=\frac{\pi^{2}}{6}-\frac{\pi \left|x\right|}{2}+\frac{x^{2}}{4}.$$

According to Lemma A1, when the variable $x_{h}$ satisfies $-2⩽x_{h}⩽2$, the series ∑m=1∞cosπmxhcosπmxhm2m2 admits a closed-form expression. Moreover, since $\cos\left(\pi mx_{h}\right)$ is periodic with 2, a modulo-2 folding operation can be applied without changing the value of the series. Specifically, for $x_{1},x_{2}\in \left[-3,3\right]$, the folded variable is defined as

(A5) $${\tilde{x}}_{h}=\left\{\begin{array}{cc} x_{h}, & \;|x_{h}|\leq 1,\\ x_{h}-2, & \;1<x_{h}\leq 3,\\ x_{h}+2, & \;-3\leq x_{h}<-1, \end{array}\right.\;h=1,2.$$

and for $x_{3},x_{4}\in \left[-2,4\right]$, the folded variable is defined as

(A6) $${\tilde{x}}_{h}=\left\{\begin{array}{cc} x_{h}, & \;|x_{h}|\leq 1,\\ x_{h}-2, & \;1<x_{h}\leq 3,\\ x_{h}-4, & \;3<x_{h}\leq 4,\\ x_{h}+2, & \;-2\leq x_{h}<-1, \end{array}\right.\;h=3,4.$$

Accordingly, (A2) can be further simplified as

(A7) $$\begin{array}{cc} \left(a\right) & =\frac{1}{2\pi^{2}}\left(\sum_{m=1}^{\infty }\frac{\cos\left(\pi m{\tilde{x}}_{1}\right)}{m^{2}}+\sum_{m=1}^{\infty }\frac{\cos\left(\pi m{\tilde{x}}_{2}\right)}{m^{2}}\right.\left.-\sum_{m=1}^{\infty }\frac{\cos\left(\pi m{\tilde{x}}_{3}\right)}{m^{2}}-\sum_{m=1}^{\infty }\frac{\cos\left(\pi m{\tilde{x}}_{4}\right)}{m^{2}}\right)\\ & =\frac{1}{8}\left(\left({{\tilde{x}}_{1}}^{2}\;+\;{{\tilde{x}}_{2}}^{2}-{{\tilde{x}}_{3}}^{2}-{{\tilde{x}}_{4}}^{2}\right)+2\left(\left|{\tilde{x}}_{3}\right|\;+\;\left|{\tilde{x}}_{4}\right|-\left|{\tilde{x}}_{1}\right|-\left|{\tilde{x}}_{2}\right|\right)\right). \end{array}$$

Substituting (A7) into (A1), the closed-form expression of the diagonal entries of $\lim_{M\to \infty }A_{i}^{H}A_{j}$ is obtained as

(A8) $$\sum_{m=-\infty }^{\infty }\alpha_{i,m}^{*}\alpha_{j,m}\;=\;\frac{1}{8}\left(\left({{\tilde{x}}_{1}}^{2}\;+\;{{\tilde{x}}_{2}}^{2}-{{\tilde{x}}_{3}}^{2}-{{\tilde{x}}_{4}}^{2}\right)+2\left(\left|{\tilde{x}}_{3}\right|\;+\;\left|{\tilde{x}}_{4}\right|-\left|{\tilde{x}}_{1}\right|-\left|{\tilde{x}}_{2}\right|\right)\right)+\tau_{i}\tau_{j}$$

Similarly, for the harmonic aliasing case with q≠0, the off-diagonal elements of $\lim_{M\to \infty }A_{i}^{H}A_{j}$ can be written as

(A9) $$\begin{array}{cc} \; & \sum_{m=-\infty }^{\infty }\alpha_{i,m}^{*}\alpha_{j,m+q}=\tau_{i}\tau_{j}\sum_{m=-\infty }^{\infty }\mathrm{sinc}\left(\pi m\tau_{i}\right)\mathrm{sinc}\left(\pi \left(m+q\right)\tau_{j}\right)e^{j2\pi m({\bar{\tau }}_{i}-{\bar{\tau }}_{j})}e^{-j2\pi q{\bar{\tau }}_{j}}\\ \; & =\frac{e^{-j2\pi q{\bar{\tau }}_{j}}}{q}\sum_{\begin{array}{c} m=-\infty \\ m\neq 0,\;m\neq -q \end{array}}^{\infty }\frac{\sin\left(\pi m\tau_{i}\right)\sin\left(\pi \left(m+q\right)\tau_{j}\right)}{\pi^{2}m}e^{j2\pi m({\bar{\tau }}_{i}-{\bar{\tau }}_{j})}\\ \; & \;-\frac{e^{-j2\pi q{\bar{\tau }}_{i}}}{q}\sum_{\begin{array}{c} m=-\infty \\ m\neq 0,\;m\neq -q \end{array}}^{\infty }\frac{\sin\left(\pi m\tau_{i}\right)\sin\left(\pi \left(m+q\right)\tau_{j}\right)}{\pi^{2}\left(m+q\right)}e^{j2\pi m({\bar{\tau }}_{i}-{\bar{\tau }}_{j})}+\tau_{i}\alpha_{j,q}+\tau_{j}\alpha_{i,q}. \end{array}$$

For convenience, introduce a new auxiliary variable t=m+q, and rewrite (A9) into a symmetric two-term structure, denoted as

(A10) $$\sum_{m=-\infty }^{\infty }\alpha_{i,m}^{*}\alpha_{j,m+q}=\left(b\right)-\left(c\right)+\tau_{i}\alpha_{j,q}+\tau_{j}\alpha_{i,q}$$

where

(A11) $$\left\{\begin{array}{cc} \left(b\right)≜\;\; & \frac{1}{q}e^{-j2\pi q{\bar{\tau }}_{j}}\sum_{\begin{array}{c} m=-\infty \\ m\neq 0,\;m\neq -q \end{array}}^{\infty }\frac{\sin\left(\pi m\tau_{i}\right)\sin\left(\pi \left(m+q\right)\tau_{j}\right)}{\pi^{2}m}e^{j2\pi m({\bar{\tau }}_{i}-{\bar{\tau }}_{j})},\\ \left(c\right)≜\;\; & \frac{1}{q}e^{-j2\pi q{\bar{\tau }}_{i}}\sum_{\begin{array}{c} t=-\infty \\ t\neq 0 \end{array}}^{\infty }\frac{\sin\left(\pi t\tau_{j}\right)\sin\left(\pi \left(t-q\right)\tau_{i}\right)}{\pi^{2}t}e^{j2\pi t({\bar{\tau }}_{i}-{\bar{\tau }}_{j})}. \end{array}\right.$$

Using Euler’s formula, the term $\left(b\right)$ in (48) can be further expressed as a linear combination of exponential series, i.e.,

(A12) $$\begin{array}{cc} \left(b\right)\; & =\frac{1}{4\pi^{2}q}e^{-j2\pi q{\bar{\tau }}_{j}}[e^{-j\pi q\tau_{j}}\left(\sum_{m\neq 0}\frac{e^{j2\pi m\left({\bar{\tau }}_{i}-{\bar{\tau }}_{j}+\frac{\tau_{i}-\tau_{j}}{2}\right)}}{m}-\sum_{m\neq 0}\frac{e^{j2\pi m\left({\bar{\tau }}_{i}-{\bar{\tau }}_{j}-\frac{\tau_{i}+\tau_{j}}{2}\right)}}{m}\right)\\ \; & \;+e^{j\pi q\tau_{j}}\left(\sum_{m\neq 0}\frac{e^{j2\pi m\left({\bar{\tau }}_{i}-{\bar{\tau }}_{j}-\frac{\tau_{i}-\tau_{j}}{2}\right)}}{m}-\sum_{m\neq 0}\frac{e^{j2\pi m\left({\bar{\tau }}_{i}-{\bar{\tau }}_{j}+\frac{\tau_{i}+\tau_{j}}{2}\right)}}{m}\right)]. \end{array}$$

and similarly,

(A13) $$\begin{array}{cc} (c)\; & =\frac{1}{4\pi^{2}q}e^{-j2\pi q{\bar{\tau }}_{i}}[e^{-j\pi q\tau_{i}}\left(\sum_{t\neq 0}\frac{e^{j2\pi t\left({\bar{\tau }}_{i}-{\bar{\tau }}_{j}+\frac{\tau_{i}-\tau_{j}}{2}\right)}}{t}-\sum_{t\neq 0}\frac{e^{j2\pi t\left({\bar{\tau }}_{i}-{\bar{\tau }}_{j}+\frac{\tau_{i}+\tau_{j}}{2}\right)}}{t}\right)\\ \; & \;+e^{j\pi q\tau_{i}}\left(\sum_{t\neq 0}\frac{e^{j2\pi t\left({\bar{\tau }}_{i}-{\bar{\tau }}_{j}-\frac{\tau_{i}-\tau_{j}}{2}\right)}}{t}-\sum_{t\neq 0}\frac{e^{j2\pi t\left({\bar{\tau }}_{i}-{\bar{\tau }}_{j}-\frac{\tau_{i}+\tau_{j}}{2}\right)}}{t}\right)]. \end{array}$$

Substituting (A12) and (A13) into (A10), the off-diagonal entries of $\lim_{M\to \infty }A_{i}^{H}A_{j}$ can be rewritten as

(A14) $$\begin{array}{cc} \sum_{m=-\infty }^{\infty }\alpha_{i,m}^{*}\alpha_{j,m+q}\; & =\frac{1j}{2\pi^{2}q}\left(\left(e^{-j\pi q\left(\tau_{j}+2{\bar{\tau }}_{j}\right)}-e^{-j\pi q\left(\tau_{i}+2{\bar{\tau }}_{i}\right)}\right)\sum_{m=1}^{\infty }\frac{\sin\left(\pi mX_{1}\right)}{m}\right.\\ \; & -\left(e^{-j\pi q\left(\tau_{j}+2{\bar{\tau }}_{j}\right)}-e^{j2\pi q\left(\tau_{i}-2{\bar{\tau }}_{i}\right)}\right)\sum_{m=1}^{\infty }\frac{\sin\left(\pi mX_{2}\right)}{m}\\ \; & +\left(e^{j\pi q\left(\tau_{j}-2{\bar{\tau }}_{j}\right)}-e^{j2\pi q\left(\tau_{i}-2{\bar{\tau }}_{i}\right)}\right)\sum_{m=1}^{\infty }\frac{\sin\left(\pi mX_{3}\right)}{m}\\ \; & \left.-\left(e^{j\pi q\left(\tau_{j}-2{\bar{\tau }}_{j}\right)}-e^{-j\pi q\left(\tau_{i}+2{\bar{\tau }}_{i}\right)}\right)\sum_{m=1}^{\infty }\frac{\sin\left(\pi mX_{4}\right)}{m}\right)+\tau_{i}\alpha_{j,q}+\tau_{j}\alpha_{i,q}. \end{array}$$

where where $X_{1},X_{2},X_{3}$, and $X_{4}$ are determined by the modulation parameters and are defined as

(A15) $$\left\{\begin{array}{c} X_{1}≜2\left({\bar{\tau }}_{i}-{\bar{\tau }}_{j}\right)+\left(\tau_{i}-\tau_{j}\right)\in \left[-3,3\right],\\ X_{2}≜2\left({\bar{\tau }}_{i}-{\bar{\tau }}_{j}\right)-\left(\tau_{i}+\tau_{j}\right)\in \left[-4,2\right],\\ X_{3}≜2\left({\bar{\tau }}_{i}-{\bar{\tau }}_{j}\right)-\left(\tau_{i}-\tau_{j}\right)\in \left[-3,3\right],\\ X_{4}≜2\left({\bar{\tau }}_{i}-{\bar{\tau }}_{j}\right)+\left(\tau_{i}+\tau_{j}\right)\in \left[-2,4\right]. \end{array}\right.$$

**Lemma** **A2.**
*Assuming that the variable x satisfies 0⩽x⩽2π, the following holds*

(A16)
$$\sum_{r=1}^{\infty }\frac{\sin\left(rx\right)}{r}=\left\{\begin{array}{cc} \frac{\pi -x}{2}, & 0<x<2\pi ,\\ 0, & x=0\;\mathrm{or}\;2\pi . \end{array}\right.$$

When the variable $X_{h}$ satisfies $0<X_{h}<2$, by letting $x=\pi X_{h}$ in Lemma A2, the series $\sum_{m=1}^{\infty }\frac{\sin(\pi mX_{h})}{m}$ admits a closed-form expression. On the other hand, since $\sin(\pi mX_{h})$ is a periodic function with period 2 with respect to $X_{h}$, the variables $X_{h}$ can be equivalently folded modulo 2 without affecting the value of the series. Specifically, for $X_{1},X_{3}\in \left[-3,3\right]$

(A17) $${\tilde{X}}_{h}=\left\{\begin{array}{cc} X_{h}+4, & -3\leq X_{h}<-2,\\ X_{h}+2, & -2\leq X_{h}<0,\\ X_{h}, & 0\leq X_{h}<2,\\ X_{h}-2, & 2\leq X_{h}\leq 3, \end{array}\right.\;h=1,3.$$

For $X_{2}\in \left[-4,2\right]$,

(A18) $${\tilde{X}}_{2}=\left\{\begin{array}{cc} X_{2}+4, & -4\leq X_{2}<-2,\\ X_{2}+2, & -2\leq X_{2}<0,\\ X_{2}, & 0\leq X_{2}\leq 2, \end{array}\right.$$

for $X_{4}\in \left[-2,4\right]$,

(A19) $${\tilde{X}}_{4}=\left\{\begin{array}{cc} X_{4}+2, & -2\leq X_{4}<0,\\ X_{4}, & 0\leq X_{4}<2,\\ X_{4}-2, & 2\leq X_{4}\leq 4. \end{array}\right.$$

Therefore, by invoking Lemma A2 and substituting the modulo-2 folded auxiliary variables ${\tilde{X}}_{1},{\tilde{X}}_{2},{\tilde{X}}_{3}$, and ${\tilde{X}}_{4}$ into (A14), the closed-form expression of the off-diagonal elements of $\lim_{M\to \infty }A_{i}^{H}A_{j}$ can be derived as

(A20) $$\begin{array}{cc} \; & \sum_{m=-\infty }^{\infty }\alpha_{i,m}^{*}\alpha_{j,m+q}=\frac{j}{2\pi^{2}q}[\left(e^{-j\pi q(2{\bar{\tau }}_{j}+\tau_{j})}-e^{-j\pi q(2{\bar{\tau }}_{i}+\tau_{i})}\right)S\left({\tilde{X}}_{1}\right)\\ \; & \;-\left(e^{-j\pi q(2{\bar{\tau }}_{j}+\tau_{j})}-e^{-j\pi q(2{\bar{\tau }}_{i}-\tau_{i})}\right)S\left({\tilde{X}}_{2}\right)+\left(e^{-j\pi q(2{\bar{\tau }}_{j}-\tau_{j})}-e^{-j\pi q(2{\bar{\tau }}_{i}-\tau_{i})}\right)S\left({\tilde{X}}_{3}\right)\\ \; & \;-\left(e^{-j\pi q(2{\bar{\tau }}_{j}-\tau_{j})}-e^{-j\pi q(2{\bar{\tau }}_{i}+\tau_{i})}\right)S\left({\tilde{X}}_{4}\right)]+\tau_{i}\alpha_{j,q}+\tau_{j}\alpha_{i,q} \end{array}$$

where the function $S\left(X\right)$ is defined as

(A21) $$S\left(X\right)=\left\{\begin{array}{cc} \frac{\pi \left(1-X\right)}{2}, & X\in \left(0,2\right),\\ 0, & X=0\;\mathrm{or}\;2. \end{array}\right.$$

Under the worst-case infinite-harmonic joint observation at Willie, the KL divergence can be reformulated into a finite-dimensional matrix expression consisting of a log-determinant term and a trace term, both of which admit closed-form matrix entries. The proof is thus completed.
